# Structure and Antigenicity of Kaposi's Sarcoma‐Associated Herpesvirus Glycoprotein B

**DOI:** 10.1002/advs.202502231

**Published:** 2025-04-26

**Authors:** Xin‐Yan Fang, Cong Sun, Chu Xie, Bing‐Zhen Cheng, Zheng‐Zhou Lu, Ge‐Xin Zhao, Sen‐Fang Sui, Mu‐Sheng Zeng, Zheng Liu

**Affiliations:** ^1^ Cryo‐electron Microscopy Center Southern University of Science and Technology Shenzhen Guangdong 518055 China; ^2^ Department of Biology Southern University of Science and Technology Shenzhen Guangdong 518055 China; ^3^ State Key Laboratory of Oncology in South China Guangdong Provincial Clinical Research Center for Cancer Guangdong Key Laboratory of Nasopharyngeal Carcinoma Diagnosis and Therapy Sun Yat‐sen University Cancer Center Guangzhou 510060 P. R. China; ^4^ Department of Dermatology Vagelos College of Physicians and Surgeons Columbia University New York NY USA

**Keywords:** antibodies, cryo‐electron microscopy (cryo‐EM), glycoprotein B (gB), kaposi's sarcoma‐associated herpesvirus (KSHV)

## Abstract

Kaposi's sarcoma‐associated herpesvirus (KSHV), a member of the human γ‐herpesviruses family, exhibits extensive cellular tropism and is associated with Kaposi's sarcoma and various B‐cell malignancies. Despite its clinical significance, no effective prophylactic vaccines or specific therapeutics are currently available to prevent or treat KSHV infection. Similar to other herpesviruses, KSHV depends on the envelope glycoprotein B (gB) for host receptor recognition and membrane fusion initiation, making gB a prime target for antiviral antibody or vaccine development. In this study, the high‐resolution cryo‐electron microscopy (cryo‐EM) structure of KSHV gB is presented, revealing a unique trimeric conformation resembling the postfusion state observed in other herpesviruses. Additionally, the structure of the non‐neutralizing monoclonal antibody 2C4 bound to KSHV gB domain IV is resolved. The comparative sequence and structure analyses reveal significant homology in neutralizing epitopes between KSHV and Epstein‐Barr virus (EBV) gB, indicating a potential pathway for the development of broad‐spectrum antiviral strategies. These findings provide a foundation for a deeper understanding of KSHV's infectious mechanism and pave the way for the creation of universal interventions against the human γ‐herpesviruses.

## Graphical abstract

Kaposi's sarcoma‐associated herpesvirus (KSHV) causes severe cancers without effective treatments. This study presents an atomic model of KSHV's critical infection protein, gB, revealing a unique structure shared with related herpesviruses like Epstein‐Barr virus (EBV). The structural similarity highlights vulnerable targets for antibodies, guiding the design of vaccines and therapies to block KSHV and potentially other human herpesviruses.

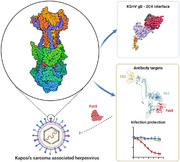

## 1 Introduction

Human herpesviruses are characterized by their double‐stranded DNA and enveloped structure, leading to lifelong latent infections. These viruses are classified into α‐, β‐, and γ‐herpesviruses.^[^ ^1^, ^2^, ^3^, ^4^, ^5^ ^]^ Notably, the γ‐herpesvirus Kaposi's sarcoma–associated herpesvirus (KSHV) is implicated in multiple malignancies, including Kaposi's sarcoma (KS), primary effusion lymphoma (PEL), and multicentric Castleman's disease (MCD).^[^ ^6^, ^7^, ^8^, ^9^ ^]^ This is particularly concerning in sub‐Saharan African countries, where KS has emerged as one of the most prevalent cancers in men and children, underscoring the global health challenge posed by KSHV.^[^ ^10^ ^]^ Despite its profound clinical impact, the development of specific antiviral treatments or vaccines targeting KSHV remains elusive.

KSHV exhibits a broad tropism for a variety of human cells, such as endothelial cells, epithelial cells, B cells, dendritic cells, and fibroblasts.^[^ ^11^ ^]^ It encodes several key envelope glycoproteins—gB, gH, and gL—that are critical for host receptor recognition, membrane attachment, and cell entry, a trait shared with other herpesviruses.^[^ ^12^, ^13^, ^14^ ^]^ These glycoproteins, particularly gB, are conserved across all herpesvirus families, functioning as a universally conserved viral fusion protein both structurally and functionally.^[^ ^15^, ^16^ ^]^ Specifically, KSHV gB is essential for viral receptor binding,^[^ ^17^, ^18^ ^]^ engages with a range of receptors across various cell types—including heparan sulfate proteoglycans (HSPGs)^[^ ^19^ ^]^; Dendritic cells‐specific intercellular adhesion molecule‐grabbing nonintegrin (DC‐SIGN) in dendritic cells, macrophages and B cells^[^ ^20^ ^]^; integrins in endothelial cells, epithelial cells and fibroblasts^[^ ^21^ ^]^; and recently, neuropilin‐1 (NRP1) in mesenchymal stem cells (MSCs).^[^ ^22^ ^]^ In addition to receptor binding, gB is crucial for viral egress,^[^ ^23^ ^]^ cytoskeletal rearrangement in host cells,^[^ ^24^, ^25^ ^]^ and cytokine secretion,^[^ ^26^ ^]^ highlighting its central role in facilitating KSHV's infection across a wide cell spectrum. As the fusion protein, gB mediates viral membrane fusion with the host membrane, triggered by signal from the gH/gL heterodimer—a mechanism consistent across herpesviruses.^[^ ^11^, ^27^, ^28^, ^29^, ^30^ ^]^ Nonetheless, the lack of detailed structural information on KSHV gB limits our understanding of its interaction mechanisms with receptor or other glycoproteins during infection and the identification of receptor bindings diversity.

The conserved nature of gB across the herpesvirus families makes it a promising target for neutralizing antibodies and vaccines development.^[^ ^31^, ^32^, ^33^, ^34^, ^35^ ^]^ Various neutralizing antibodies against gB have been successfully identified in the context of herpes simplex virus 1 (HSV‐1),^[^ ^36^ ^]^ varicella‐zoster virus (VZV),^[^ ^37^ ^]^ human cytomegalovirus (HCMV),^[^ ^38^, ^39^ ^]^ and Epstein‐Barr virus (EBV),^[^ ^40^ ^]^ showcasing gB's potential as a viable antigen for novel prophylactic vaccine initiatives.^[^ ^41^, ^42^ ^]^ Moreover, recent studies have identified a conserved antigenic domain within gB that maintains its exposed antigenic site across both prefusion and postfusion conformations, a characteristic attributed to gB's structure and sequence homology across herpesviruses.^[^ ^36^ ^]^ This pivotal discovery opens new pathways for the exploration of KSHV‐specific antiviral therapies and vaccine research.^[^ ^36^ ^]^ Consequently, a thorough investigation into KSHV gB's vaccine target potential, particularly its antigenic epitope capable of facilitating cross‐herpesvirus recognition, is essential. Determining the structure of KSHV gB and comprehending how herpesvirus‐specific gB antibodies interact with it are critical steps forward in these research endeavors.

In this study, we resolved the cryo‐electron microscopy (cryo‐EM) structure of KSHV gB ectodomain, achieving a resolution of 3.26 Å, enabling the construction of an atomic model. This structure reveals a classic trimeric structure in its postfusion conformation, aligning with those observed in other herpesviruses and affirming its categorization as a class III fusion protein. In addition, we resolved the structure of the non‐neutralizing monoclonal antibody (mAb) 2C4 bound to KSHV gB domain IV (DIV). Through sequence and structure comparisons, we identified a significant homology between KSHV gB and that of EBV, underscoring a close evolutionary relationship. Parallel experiments involving herpesvirus gB antibody binding assay and sub‐domain antibody footprint alignment further demonstrated the cross‐recognition of the EBV neutralizing antibody toward KSHV gB, indicating shared neutralizing epitopes among γ‐herpesviruses. These critical insights lay the groundwork for understanding KSHV's receptor binding and fusion process, while also supporting the potential development of targeted vaccines against KSHV and possibly other members of the γ‐herpesvirus family.

## 2 Results

### 2.1 Cryo‐EM Structure of KSHV gB Ectodomain in Postfusion Conformation

**Figure 1:**
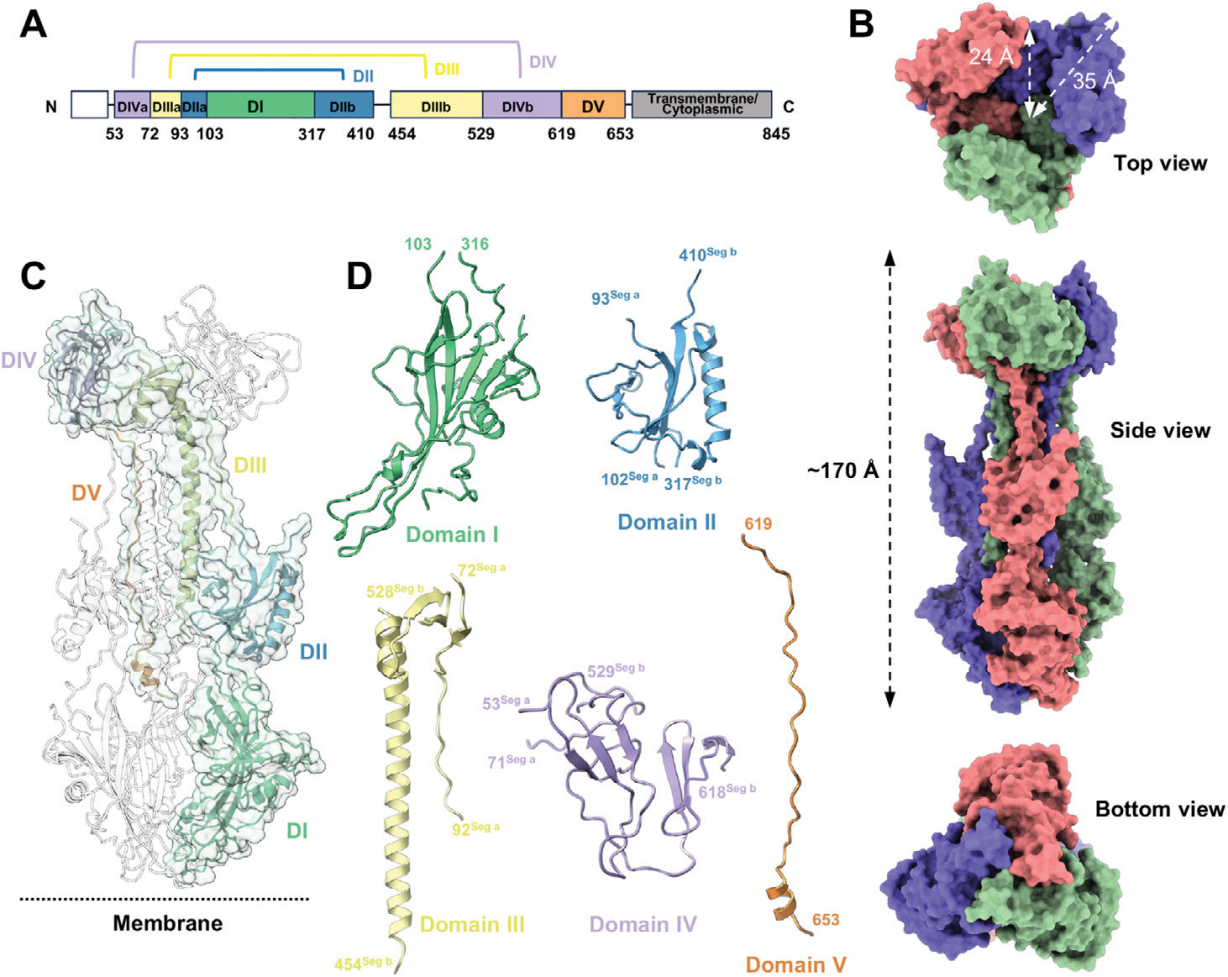
Structure of KSHV gB ectodomain in postfusion conformation. A) Sequence and domain segmentation of full‐length KSHV gB. Domains are highlighted in different colors and the intervening sites are marked. B) Structural overview of KSHV gB trimer. Each protomer is colored differently, and three distinct views (side, top, and bottom) are presented. C) The density map and structural overview of KSHV gB. D) Domain details of KSHV gB. The KSHV gB monomer is depicted as a cartoon, with each domain colored differently. The starting and ending residues of each domain and segment are labeled. Structure of KSHV gB trimer displayed as a cartoon. The gB monomer domains are highlighted in colors consistent with (C). The N‐ and C‐terminus, the location FL, and suspected orientation toward the membrane are indicated.

The structural elucidation of the KSHV gB ectodomain was achieved using single‐particle cryo‐EM, attaining an overall resolution of 3.26 Å (Table S1, Supporting Information). The KSHV gB653‐mut ectodomain, analyzed in our study, adopts the well‐characterized postfusion gB structure, forming a homo‐trimeric, rod‐like spike. This structure measures ≈170 Å in height, with a major and minor radius of ≈35 Å and 24 Å, respectively (**Figure** **1**B). The structure consists of five distinct domains (Domain I through V or DI‐DV), spanning residues 53–653. However, a certain region (411–453) was unresolved in the cryo‐EM map (Figure 1A).

The N‐terminus of KSHV gB originates from DIVa, extends through DIIIa and DIIa to the lower fusion loop (FL) I DI in the bottom, wraps back to DIIb, DIIIb, toward to the top end in DIVb, and ends in DV inside, forming a palindromic arrangement of subdomains within the trimeric structure (Figure 1C,D). We identified three pairs of intramolecular disulfide bonds (C^68^‐C^528^ in DIV, C^85^‐C^484^ in DIII, and C^315^‐C^362^ in DII) and observed no inter‐chain disulfide bonding, indicating a tight but flexible trimeric assembly.

**Figure 2:**
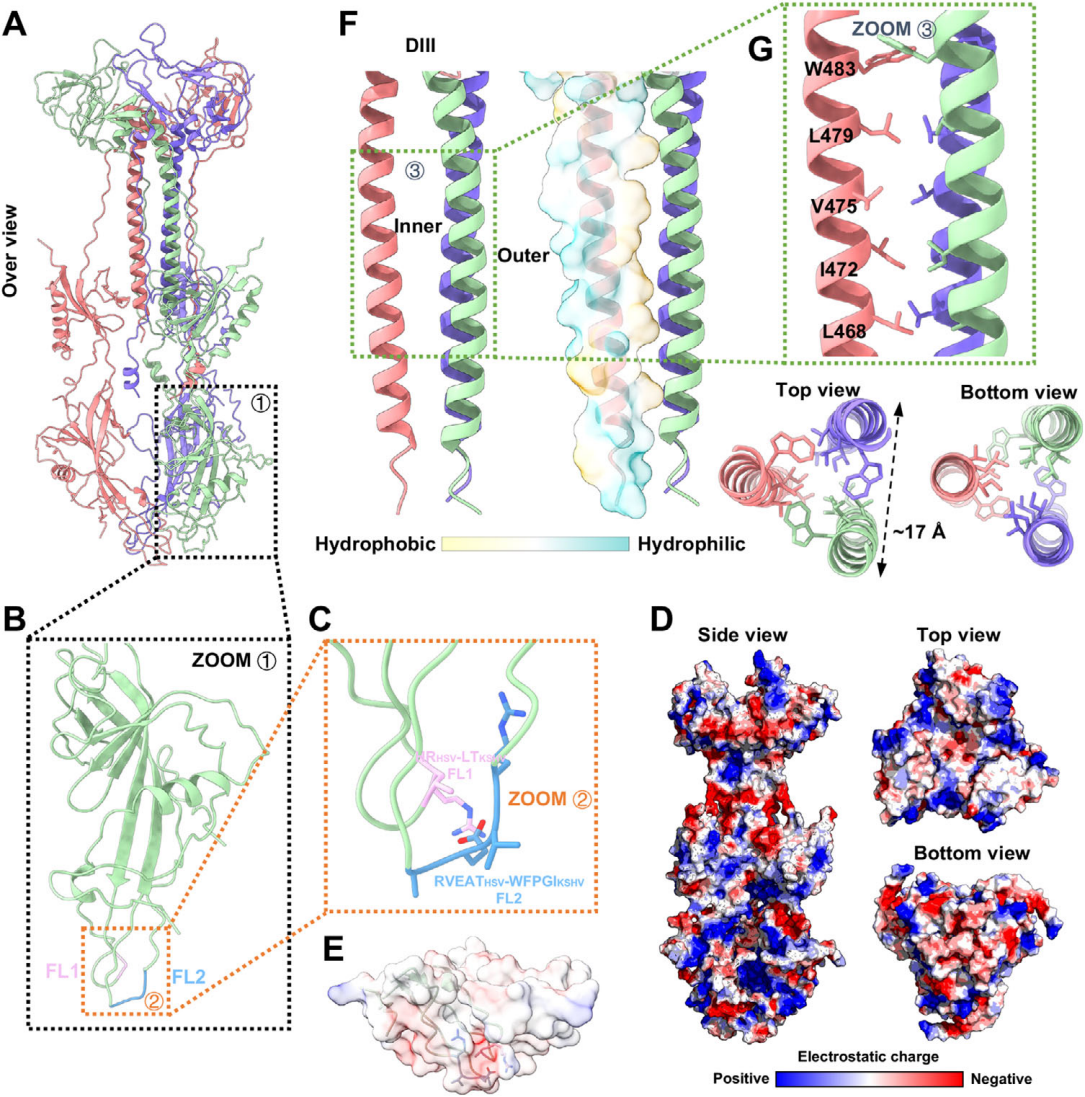
Sub‐structure details of KSHV gB mutated FL, electrostatic surface, and trimerization. A) Architecture of KSHV gB653‐mut ectodomain trimer. B) Details for Domain I. C) Details for FL. The FL are located at the bottom of Domain I, protruding from the strands and the FL is replaced by LT‐FL1 and WFPGI‐FL2 in HSV‐1. D) The electrostatic distribution of KSHV gB is shown from different perspectives. The charges are displayed as red (negative), white (neutral), and blue (positive), as indicated by the scale bar. E) Electrostatic distribution of the FL of (B). F) The trimeric helices of Domain III are depicted in a cartoon scheme on the left, and the hydrophilicity‐hydrophobicity map is shown on the right. G) Details of the hydrophobic tunnel inside the Domain III helical bundle, composed of residues L468, I472, V475, L479, and W483. These residues are presented as sticks, colored consistently with the hydrophobicity calculated in three different perspectives (side, top, and bottom) are presented.

Reflecting the high degree of homology across herpesvirus gBs, the atomic model of KSHV gB exhibits similar domain organizations with distinctive features (**Figure** **2**A; Figure S3, Supporting Information). Specifically, Domain I (DI) spans from K^103^ to P^316^ and incorporates a pleckstrin‐homology (PH) domain and anti‐parallel strands that are located on top of two FL, FL1 and FL2, forming the base of the trimer (Figure 2B,C; Figure S5, Supporting Information). The native FL have been associated with protein aggregation and reduced yield in EBV gB expression,^[^ ^34^ ^]^ similarly, the native FL were suspected to contribute to the unsuccessful expression of the wild‐type KSHV gB ectodomain (Figure S1, Supporting Information). The FLs of the wild‐type KSHV gB consist of two loops, LT‐FL1 and WFPGI‐FL2, characterized by high hydrophobicity (L‐FL1; W, F, P, G, I‐FL2), whereas the KSHV gB653‐mut FL, HR‐FL1 and RVEA‐FL2 from HSV‐1 gB, incorporate with polar amino acid (H, R‐FL1; R, E, T‐FL2). This variation in amino acid characteristics within the FL has also been observed in EBV gB, where the replacement of the wild‐type's hydrophobic WY‐FL1/WLIW‐FL2 (W‐FL1; W, L, I, W‐FL2) with HSV‐1′s HR‐FL1 and RVEA‐FL2 occurred. These substitutions result in an overall electrostatic charge map revealing a positively charged head and a negatively charged bottom (Figure 2G). In detail, the presence of acidic (E^211^‐FL2) and basic amino acids (R^129^‐FL1; R^209^‐FL2) renders the lowest bottom of KSHV gB653‐mut highly hydrophilic, with a negatively charged bottom tip due to the carboxyl group of glutamic acid, surrounded by positively charged side chains from the amidogen of arginine. This may stabilize the gB653‐mut ectodomain in a solvent environment during purification, compared to the wild‐type construct (Figure 2D).

Domain II (DII) consists of two parts, DIIa (H^93^‐K^102^) and DIIb (L^317^‐T^410^), also includes a PH domain, intersected by six strands and an outer helix, connecting the third and fourth strands (Figure 1D). Typically, the PH domain acts as a scaffold, facilitating cytoplasmic signaling pathways and enabling phosphoinositide or protein‐ligand binding. In this context, DI and DII, with their PH domains, may play a pivotal role in recognizing host receptors or receiving fusion triggering signals from other glycoproteins such as gH/gL.

Domain III (DIII) also consists of two parts, DIIIa (I^72^‐ Y^92^) and DIIIb (D^454^‐C^528^), featuring a central, long helix forming a trimeric, left‐handed bundle, providing strong interchain contacts (Figure 2E,F; Figure S3, Supporting Information). Examination of the bundle revealed that the outer and inner surfaces of the helix display distinct hydrophilic characteristics, with the inner surface being highly hydrophobic. The residues L^468^, I^472^, V^475^, L^479^, and W^483^, located inside the bundle, form a trimeric hydrophobic tunnel, further constrained by the exterior hydrophilic surface of the helix (Figure 2G).

Domain IV (DIV) consists of two parts, DIVa (E^53^‐ S^71^) and DIIIb (I^529^‐I^618^), positioned at the trimer's apex, is distinguished by its looped structure and a critical disulfide bond linking two segments, while DV extends downward, suggesting potential roles in stabilizing the trimeric assembly through interactions with DIII's central helices (Figure 1D; Figure S3, Supporting Information). A disulfate bond between C^68^‐C^528^ connected DIVa and DIVb.

Domain V (DV) extends downward from the C‐terminus of DIV in a long loop, ending in a short helix and seamlessly transitioning to the transmembrane domain, which remains unresolved in this structure. Although DV is distanced from the other domains within the same protomer, its loop extends parallel to the central helices of DIII from the other two protomers, potentially reinforcing the trimerization of DIII (Figure 1D; Figure S3, Supporting Information).

Despite modifications to prevent furin cleavage at the furin site, the corresponding structural region remained unresolved, likely due to inherent flexibility common in herpesvirus gB structures.

### 2.2 Cryo‐EM Structure of KHSV gB in Complex with Non‐Neutralizing mAb 2C4

To identify neutralizing epitopes on KSHV gB, we screened a previously established phage display library to screen for human monoclonal antibodies against KSHV gB.^[^ ^43^ ^]^ This screening yielded mAb 2C4, which was assessed for its binding affinity to KSHV gB, and its potential to neutralize KSHV infection. mAb 2C4 is the first human antibody against KSHV gB, providing a valuable tool for studying KSHV neutralizing mechanism, despite its lack of neutralizing activity (Figure S6A, Supporting Information).

**Figure 3:**
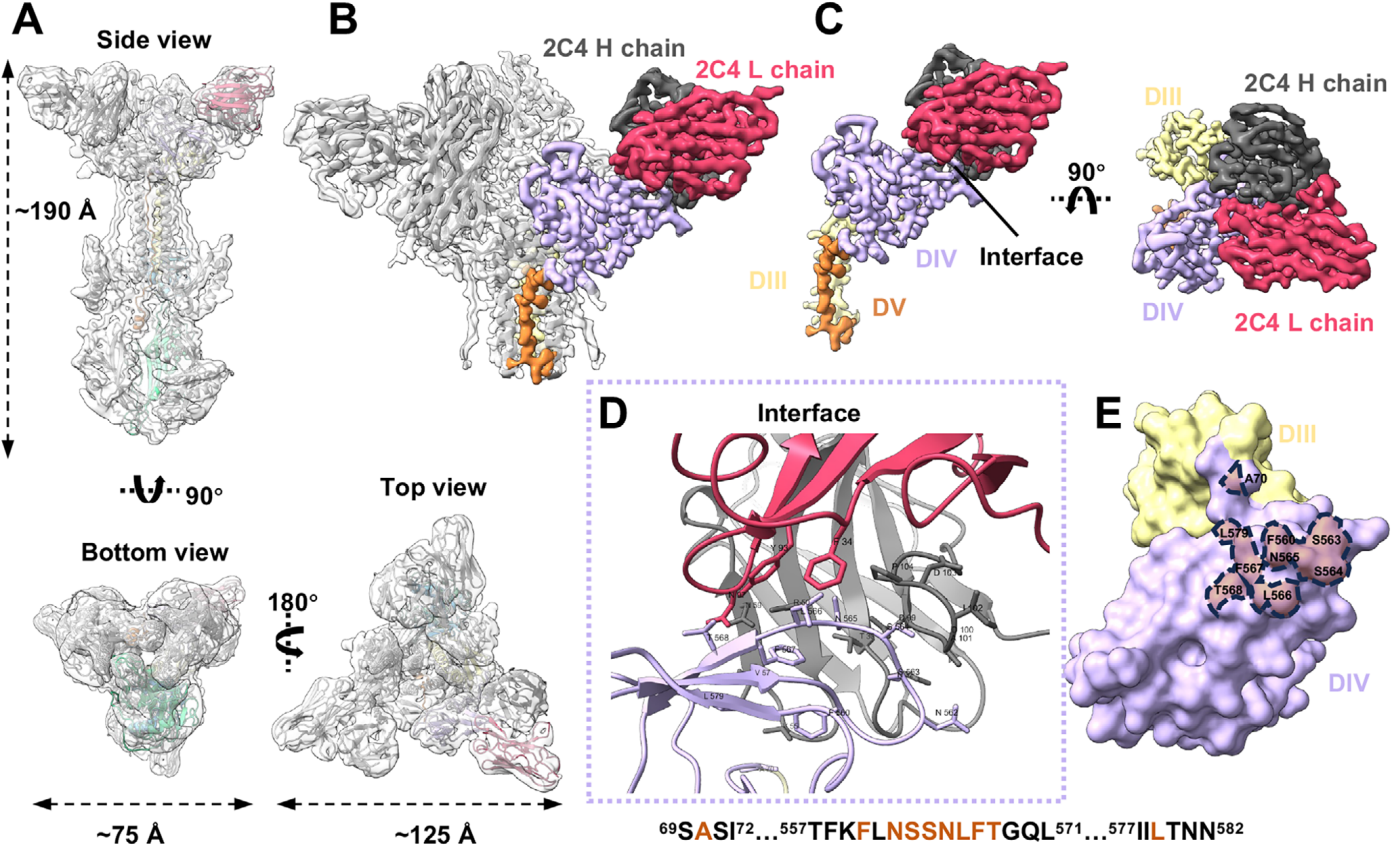
Structure of KSHV gB ectodomain in complex with mAb 2C4 Fab. A) Side, top, and bottom views of KSHV gB‐2C4 Fab map superimposed with its atomic model, represented as a ribbon. B) Local map of KSHV gB DIV‐2C4 Fab with its atomic model depicted as a cartoon. Domain III (yellow), DIV (plum), DV (dark orange) of gB and 2C4 Fab heavy chain (dim grey) light chain (Indian red) are highlighted in distinct colors for clarity. C) The interface of KSHV gB DIV‐2C4 Fab in a single monomer. D) Detailed interactions of the interface described in (C). E) The 2C4 Fab footprint on KSHV gB is outlined with dash lines.

To further investigate the antigenic properties of KSHV gB, we purified the 2C4 Fab‐KSHV gB complex (Figure S6B and S6C, Supporting Information). Using single particle cryo‐EM analysis, we determined the structure of KSHV gB in complex with mAb 2C4 at the resolution of 3.6 Å (Figure S7B, Supporting Information). Local refinement improved the resolution of the gB‐2C4 interface to 2.92 Å (Figure S7C, Supporting Information). This complex structure measures ≈190 Å in height, with a top view width of 125 Å and a bottom view width of 95 Å (**Figure** **3**A). 2C4 Fab binds to the DIV loop region of KSHV gB, with a total buried surface area (BSA) of 792.3 Å^2^ engaging both antibody's heavy chain (579.3 Å^2^) and light chain (213 Å^2^) (Figure 3B,C). PISA interface analysis identified five DIV residues interacting with seven residues of 2C4 Fab. Specifically, four hydrogen bonds (S564‐D99, S563‐N32, S564‐I102, and L566‐R50) form at the gB‐H chain interface, while four hydrogen bonds (T568‐Y93, T568‐N97, N565‐Y99, and N568‐N97) form at the gB‐L chain interface (Figure 3D,E). Previous structural studies of other herpesvirus gB‐antibody complexes have shown that several antibodies also target DIV (Figure S8, Supporting Information), including neutralizing antibodies (VZV 93K^[^ ^37^ ^]^ and EBV 3A5^[^ ^40^ ^]^), and non‐neutralizing antibodies (VZV SG2,^[^ ^34^ ^]^ HSV‐1 BMPC‐23,^[^ ^44^ ^]^ and HSV‐1 HSV010‐13^[^ ^44^ ^]^). The previous studies on these gB‐targeting neutralizing antibodies had suggested that epitope exposure and antibody binding orientation would influence their ability to target prefusion state gB to neutralize virus infection (see more in Discussion section). Due to lack of KSHV gB structure in prefusion state and other KSHV gB‐targeting antibodies structure, the reason for 2C4's lack of neutralizing activity is hard to explain.

### 2.3 Sequence and Structural Homology Analysis of Herpesvirus gB Proteins

**Figure 4:**
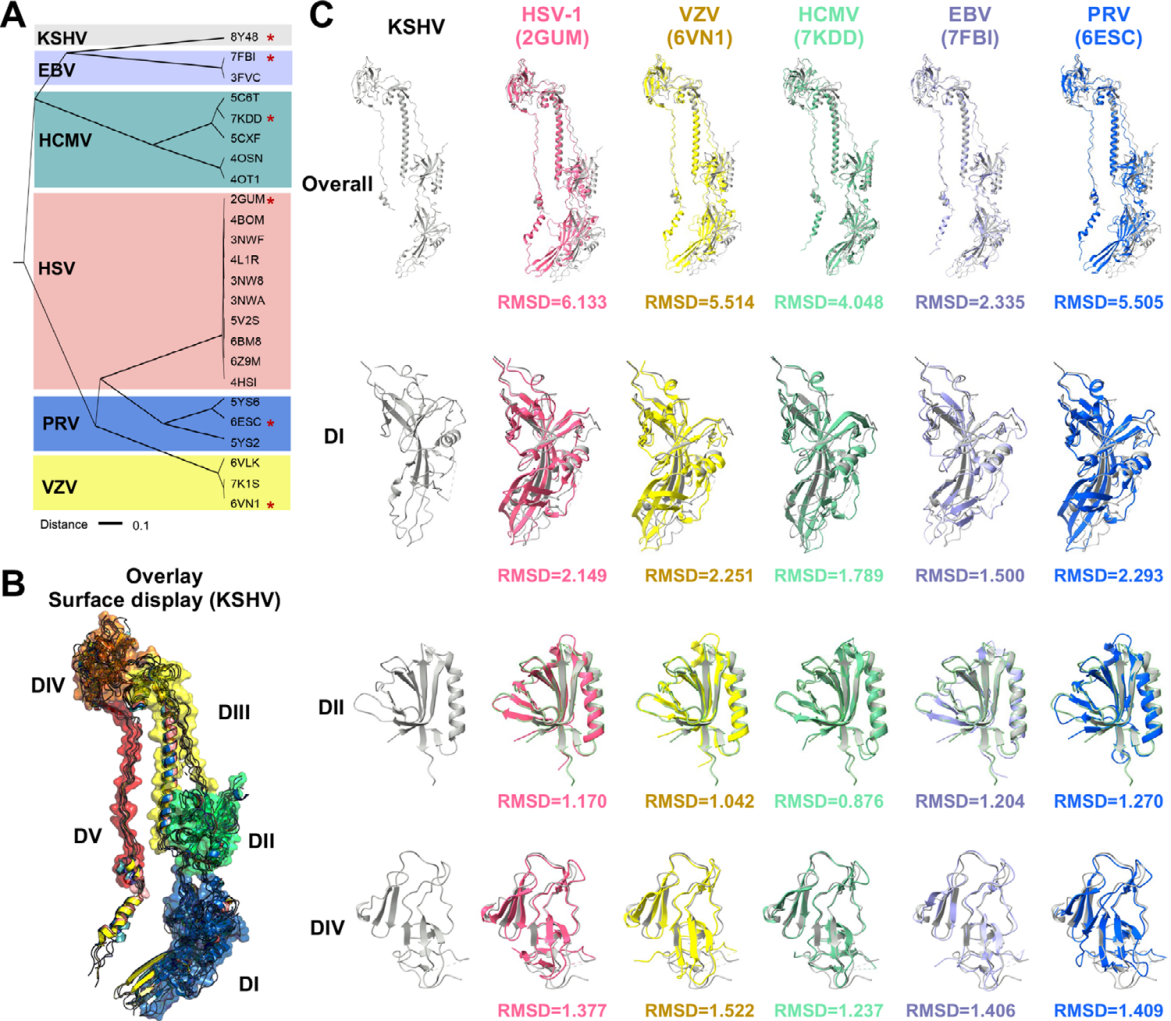
Sequence alignment, phylogenetic analysis, and structural comparison of KSHV gB with other herpesviruses gBs. A) Phylogenetic tree showing all herpesvirus gB proteins with available structural information. The tree is annotated with herpesvirus type labels, and branches are color‐coded. The distance between branches is indicated. B) Superimposition of KSHV gB and HSV‐1, VZV, HCMV, EBV, or PRV based on DIII alignment. The surfaces annotated with gB domains in the overlay structures were colored according to KSHV gB. C) Comparison of DI, DII, and DIV between KSHV and HSV‐1, VZV, HCMV, EBV, or PRV.

Since we were unable to identify a neutralization antibody against KSHV gB, we explored alternative strategies to combat KSHV infection. Sequence and structure homology between viruses often correlates with shared antigenicity, facilitating cross‐recognition by antibodies. To enhance our homology analysis in both sequence and structure levels, we used ENDscript2.X to align all gB sequences with reported structures via Clustal Omega, generating a phylogenetic tree (**Figure** **4**A). This analysis confirmed that KSHV gB is most closely related to EBV gB, followed by HCMV gB, while HSV‐1 gB is the most distantly related, consistent with our sequence‐based findings.

To expand our investigation to structural comparisons, we selected representative gB structures from HSV‐1 (PDB: 2GUM), VZV (PDB: 6VN1), HCMV (PDB: 7KDD), EBV (PDB: 7FBI), and PRV (PDB: 6ESC) for comparison with KSHV gB characterized in this study. These gB sequences displayed high similarity (Figure S9, Supporting Information). The structural alignment and subsequent model fitting revealed that KSHV gB aligns most closely with EBV gB, with the lowest root mean square deviation (RMSD) value of 2.335. This was followed by HCMV gB with an RMSD of 4.048, and HSV‐1 gB exhibiting the highest RMSD of 6.133 (Figure 4B,C). We further compare the RMSD between domains which shows the slight difference (Figure 4C). This pattern of structural similarity, consistent with the sequence homology results, strongly indicates that KSHV gB shares a closer structural and sequential resemblance with γ‐herpesvirus gB, particularly EBV gB, than with β‐ or α‐herpesvirus.

### 2.4 Cross‐Herpesviruses Antigenicity of gB Proteins

**Figure 5:**
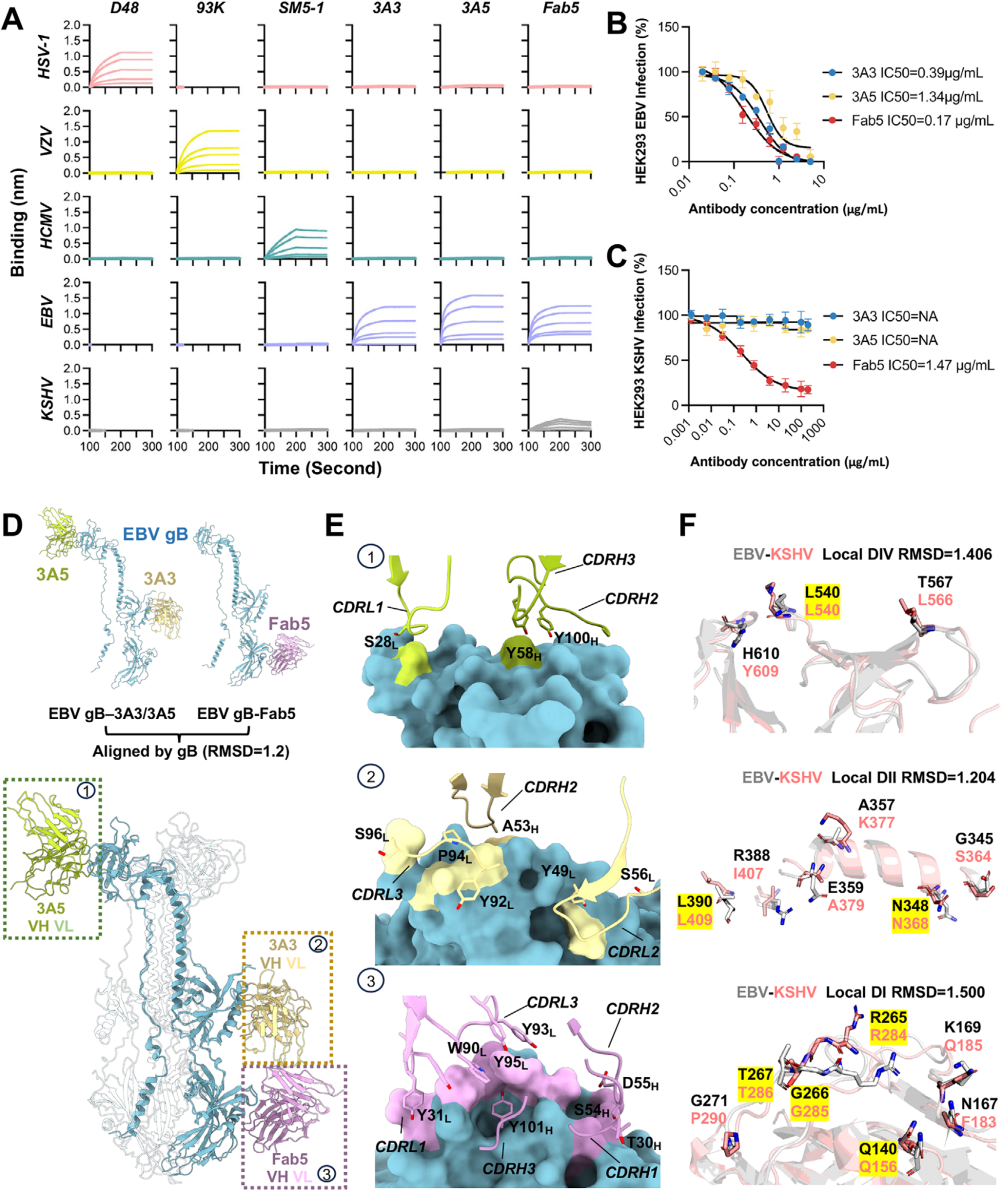
Antibody Binding profiling, cross‐neutralization, and structural alignment reveal cross‐herpesvirus antigenicity between KSHV and EBV. A) Biolayer interferometry assays were conducted using antibodies D48, 93K, SM5‐1, 3A3, 3A5, and Fab5 to measure their binding to gB proteins from HSV‐1, VZV, HCMV, EBV, and KSHV. Each graph shows the binding signal of 500, 250, 125, 62.5, and 31.25 nm (from higher to lower curves) of gB associating with an equal amount of ligand antibody loaded onto the Protein A biosensor. Data from one experiment was presented and validated by another independent replicate experiment. B) Antibody neutralization assays for EBV infection in HEK293 cells. Normalized infection rate (%) was calculated by dividing the GFP + % of each well by the mean GFP + % of the control IgG group. Non‐linear fitting (four parameters) was performed to calculate the IC50 in Prism 10.0. Data from one experiment data containing four replicate wells was presented and validated by another independent replicate experiment. C) Antibody neutralization assays for KSHV infection in HEK293 cells. The analysis protocols were same as (B). D) Superimposition of the 3A3/3A5‐EBV gB complex and the Fab5‐EBV gB complex based on alignment of gB. The fitting RMSD of the complex is 1.2. At the top: individual structures of EBV neutralizing antibodies in complex with gB. At the bottom: the ribbon structure post‐alignment. gB is colored in dark grey. The heavy (VH) and light (VL) chains of each antibody are colored differently. E) Antibody footprints of 3A3, 3A5, and Fab5 on EBV gB. The CDR loops of the antibodies are shown as ribbons, and EBV gB is shown as a surface. The colors used for the footprints on gB correspond to those of the interacting CDR residues. Labels for detailed CDR regions and the key antibody‐interacting residue names within the CDRs are provided adjacent to their respective areas. F) Key conserved residues between KSHV gB and EBV gB for cross‐recognition antibodies. EBV gB and KSHV gB are displayed as grey and pink ribbon respectively. The exact interacting residues of EBV gB with antibodies, consistent with the footprints in (E), are marked in black. The counterpart key residues, potentially involved in antibody interaction of KSHV gB based on the alignment, are marked in pink. Sites where KSHV and EBV residues are identical are highlighted with a yellow background.

Based on the high sequence similarity and gB structural homology, our study explored the cross‐species conservation between KSHV gB and EBV gB to identify vulnerable sites on KSHV gB that might be targeted by neutralizing antibodies (nAbs) for potential cross‐herpesvirus antigenicity. We purified various gB‐specific nAbs and performed parallel antibody binding assays to map the cross‐herpesvirus antigenicity profile (**Figure** **5**A; Table S2, Supporting Information). While gB‐specific antibodies targeting HSV‐1, VZV, HCMV, and EBV generally exhibited limited cross‐reactivity with gB from other herpesviruses, the newly identified EBV gB‐specific antibody Fab5 (PDB: 8YY6) demonstrated a significantly higher binding capacity to KSHV gB, followed by the HCMV gB‐specific antibody SM5‐1,^[^ ^45^ ^]^ and two other EBV gB nAbs, 3A3 and 3A5.^[^ ^40^ ^]^ This suggests that KSHV gB's antigenicity, closely related to its homology with EBV gB, provides a pathway for cross‐herpesvirus vaccine development.

To verify whether the observed antibody binding translates into cross‐ neutralization of KSHV infection, we performed virus neutralization assays with antibodies 3A3, 3A5, and Fab5 against EBV and KSHV in HEK293 epithelial cells. Remarkably, all three nAbs effectively inhibited EBV infection, with IC_50_ values of 0.39 µg mL⁻^1^ (3A3), 1.34 µg mL⁻^1^ (3A5), and 0.17 µg mL⁻^1^ (Fab5) (Figure 5B,C). However, only Fab5 which had the highest binding affinity for KSHV gB showed partial neutralization of KSHV infection, with an IC_50_ of 1.47 µg mL⁻^1^, while 3A3 and 3A5 exhibited no detectable neutralizing activity against KSHV. This outcome further implies the existence of cross‐herpesvirus vulnerable epitopes between EBV and KSHV.

To elucidate the structural basis for the cross‐recognition of KSHV gB by EBV‐specific nAbs, we analyzed co‐structures of antibody‐gB complexes, including the Fab5‐EBV gB structure (PDB: 8YY6) (Figure 5D). These structures were aligned with the EBV gB in complex with nAbs 3A3 and 3A5 (PDB: 7FBI) as a reference. Subsequently, the recombinantly aligned structure was further aligned with KSHV gB to identify potential cross‐reactivity sites (Figure 5F). The analysis revealed that the three nAbs target distinct epitopes in EBV gB, located on domains DI (Fab5), DII (3A3), and DIV (3A5). Both the complementarity‐determining regions of the heavy chains (CDRH) and light chains (CDRL) were involved in the interaction, underscoring the specificity and complexity of the antibody‐gB binding dynamics. Regarding the specifics of the polar interaction network, in antibody 3A5, S^28^ from CDRL1, Y^58^ from CDRH2, and Y^100^ from CDRH3 interacted with T^567^, H^610^ and L^540^ within the DIV domain of EBV gB, respectively. In antibody 3A3, A^53^ from CDRH2, Y^49^ and S^56^ from CDRL2, and Y^92^, P^94^, and S^96^ from CDRL3 interacted with A^357^, N^348^, G^345^, E^359^, R^388^, and L^390^ within DII domain, respectively. In antibody Fab5, T^30^ from CDRH1, S^54^ and D^55^ from CDRH2, Y^101^ from CDRH3, Y^31^ from CDRL1, and W^90^, Y^93^ and Y^95^ from CDRL3 interacted with EBV gB Q^140^, N^167^, K^169^, G^271^, T^267^, G^266^, and R^265^ in DI domain, respectively (Figure 5E; Figure S10, Supporting Information). Notably, the modified regions of KSHV gB653‐mut ectodomain are not involved in any of interaction interfaces between gB and antibodies.

The polar network plays a significant role in stabilizing the protein complex suggesting that the conservation of key interacting amino acids between EBV and KSHV is crucial for cross‐recognition. Given the observed variations in the structural similarity of domains across herpesviruses, we performed individual alignments of KSHV gB domains with their EBV counterparts. This approach improved structural alignment efficiency, facilitating the precise identification of conserved key amino acids in KSHV gB that are critical for cross‐recognition by EBV gB‐specific nAbs (Figure 5F; Figure S11, Supporting Information). For the binding of antibody 3A5, the local alignment of the EBV and KSHV gB DIV domains achieved a fit with a RMSD of 1.406. This alignment revealed that only one KSHV gB amino acid, L^540^, matched the critical interacting residues, with a hit rate of 1 out of 3. In the case of antibody 3A3, the DII domain's local alignment resulted in a fit with an RMSD of 1.204(Figures 4C and 5F, Supporting Information), showing that KSHV gB amino acids N^368^ and L^409^ corresponded to EBV gB's N^348^ and L^390^ as interacting amino acids, achieving a hit rate of 2 out of 6. However, for Fab5 binding, the DI domain's local alignment demonstrated the best fit among the comparisons with an RMSD of 1.500 (Figures 4C and 5F). It showed that four KSHV gB amino acids, Q^156^, R^284^, G^285^, and T^286^, matched their EBV gB counterparts, yielding the highest hit rate of 4 out of 7. These findings explain Fab5's strongest cross‐recognition of KSHV gB and suggest that KSHV and EBV gB share a high degree of cross‐herpesvirus antigenicity, which appears to be most pronounced in the DI.

## 3 Discussion

KSHV is the causative pathogen for KS and is also associated with a variety of human diseases and malignancies.^[^ ^46^, ^47^ ^]^ Despite its clinical significance, there are currently no available drugs or vaccines targeting KSHV, highlighting the urgent need for both fundamental research into its mechanisms of infection and oncogenesis, and translational studies for developing novel medications and vaccines.^[^ ^48^ ^]^ As a member of herpesvirus family, KSHV relies on its glycoproteins for crucial processes such as host receptor recognition and cell entry, making them prime targets for antiviral interventions. Among these, KSHV gB plays a critical and irreplaceable role during viral infection. It recognizes multiple receptors, allowing broad host cell tropism for KSHV, and collaborates with other glycoproteins such as gH/gL, to facilitate membrane fusion, a key step in viral entry. Due to its irreplaceable role, it has garnered significant attention to screen neutralizing antibodies. Elucidating the structure of KSHV gB and its epitope is fundamental to understanding viral infection processes and interactions with the host.^[^ ^49^ ^]^ Unraveling the structural details of gB and gB‐mAb complex could provide valuable insights into its functional mechanisms, potentially leading to the development of novel therapeutic strategies targeting KSHV infection.

We determined the cryo‐EM structure of the KSHV gB ectodomain at a resolution of 3.26 Å. This breakthrough was made possible by a novel construct, gB653‐mut, which incorporates strategic substitutions from the FL from HSV‐1 gB and mutations in the furin site. These modifications addressed challenges related to the lower expression level of the wild‐type ectodomain. The resolved structure of KSHV gB closely resembles the postfusion conformation observed in gB proteins across various herpesviruses, including HSV‐1, HCMV, and EBV. This finding highlights the conserved role of gB in the infection process across different herpesvirus. Notably, a similar strategy involving the substitution of HSV‐1 FL was employed to purify the soluble ectodomain of EBV gB. However, unlike KSHV and EBV gB, the gB proteins of other α‐ and β‐herpesviruses did not require such modifications for successful expression. This highlights the unique characteristics of the FL in γ‐herpesviruses, which may affect gB hydrophilicity and its interaction with cell membranes.

The previous studies had classified KSHV as a γ‐herpesvirus, similar to EBV.^[^ ^11^, ^50^ ^]^ However, the sequence and structure homology of their gB proteins, which supports this classification, remains uncertain.^[^ ^18^ ^]^ Sequence alignment confirmed a high degree similarity between KSHV and EBV gB proteins among human herpesvirus, and direct structural alignment revealed an even stronger homology, supporting a structural basis for common receptor recognition and shared immune epitopes. Previous studies have identified NRP1 as a shared gB‐binding receptor for both EBV and KSHV^[^ ^22^, ^51^ ^]^; however, the subsequent entry processes diverge based on the NRP1‐interacting internalization‐mediating factors: EGFR for EBV and PDGFR for KSHV.^[^ ^22^, ^51^ ^]^ The reported structure of KSHV gB offers crucial molecular insights, highlighting residue‐level similarities and differences that contribute to the shared NRP1 binding and the distinct interactions with host factors. Understanding the structural and functional aspects of KSHV gB can provide valuable insights into viral entry mechanisms and guide the development of targeted therapeutic interventions.

To explore potential therapeutics and effective vaccines, we screened human monoclonal antibodies against KSHV gB from a previously established phage display library. We identified mAb 2C4, the first human antibody anti‐KSHV gB. However, mAb 2C4 did not exhibit neutralizing activity. To further elucidate the binding mechanism and define its potential role, we also resolved KSHV gB‐2C4 mAb structure at an overall resolution of 3.6 Å and local interface resolution of 2.92 Å. Structural analysis revealed that mAb 2C4 bound to KSHV gB DIV, the same region targeted by other five herpesvirus gB antibodies (VZV 93K, EBV 3A5, VZV SG2, HSV‐1 BMPC‐23, and HSV‐1 HSV010‐13). Of these mAbs targeted gB DIV, four out of six are non‐neutralizing antibodies (SG2, BMPC‐23, HSV010‐13, and 2C4). Structural homology alignment revealed that EBV 3A5 and VZV SG2 share partially overlapping epitopes with KSHV 2C4. The neutralization efficacy of an antibody depends on its ability to block gB‐mediated fusion or, potentially, receptor engagement. The previous studies on these gB‐targeting neutralizing antibodies had suggested that factors such as epitope exposure and antibody binding orientation influence their ability to target gB in its prefusion state and thereby neutralize virus infection. E.g., SG2,^[^ ^34^ ^]^ a VZV gB‐targeting antibody, shares an epitope that partially overlaps with 2C4, according to structural alignment. SG2 failed to neutralize VZV infection, likely due to its epitope being inaccessible in the prefusion state of VZV gB, whereas the neutralizing antibody 93K binds an accessible site and effectively blocks fusion.^[^ ^37^ ^]^ We attempted to use AlphaFold3 for predicting KSHV gB in a prefusion state, however, the prediction did not produce a reliable prefusion model due to the limited availability of homologous structures in the current PDB database. A previous study on HCMV gB demonstrated that domains I through IV undergo extensive rearrangement, while each domain largely retains its structural fold.^[^ ^45^ ^]^ Given the high sequence and structural homology between KSHV and HCMV, we aligned the interacting region of KSHV gB‐mAb 2C4 with two HCMV prefusion structures (PDB: 7KDP^[^ ^45^ ^]^ and PDB: 8VYN^[^ ^52^ ^]^), as shown in Figure S12 (Supporting Information). Our analysis revealed that in the prefusion state, the interacting regions of KSHV gB DIV are partially obscured by adjacent gB domains. Notably, subtle discrepancies in the prefusion gB conformation have been reported across different studies (Figure S12B and S12C, Supporting Information). Hence, the lack of a resolved prefusion KSHV gB structure and additional KSHV gB‐targeting antibodies for comparative analysis prevent us from identifying the precise reason behind 2C4's lack of neutralizing activity.

Sequence and structure homology between viruses often correlates with shared antigenicity, enabling cross‐recognition by antibodies. This phenomenon, observed in various viruses such as coronaviruses and influenza, lays the groundwork for developing common vaccines and antiviral drugs.^[^ ^53^, ^54^, ^55^, ^56^, ^57^ ^]^ Given the absence of available antibodies or vaccines against KSHV, the potential cross‐recognition by antibodies targeting gB of other herpesviruses, especially EBV, offers significant promise in addressing this urgent need. In our study, a parallel antibody binding assay revealed that a novel EBV gB neutralizing antibody Fab5, targeting on DI, displayed certain degree of cross‐recognition to KSHV gB, endowing the antibody with moderate neutralizing capacity against KSHV infection. This discovery highlights the presence of common epitopes between EBV and KSHV. It also emphasized the potential conserved function of DI in γ‐herpesvirus infection. In light of successful developments in vaccine candidates and novel neutralizing antibodies for EBV based on gB,^[^ ^58^ ^]^ the availability of expression constructs and the ability to induce targeted neutralizing antibodies could significantly propel vaccine and antibody therapy development for KSHV.

In conclusion, the structure of KSHV gB ectodomain and KSHV gB‐2C4 mAb reported in this study provide a crucial foundation for advancing KSHV research. These findings aid in understanding host receptor recognition, identifying taxonomy and homology, and developing vaccine for KSHV. The significant homology between KSHV and EBV gB, both in sequence and structure, suggests a common receptor and shared antigenicity. This finding implies that vaccine development strategies used for EBV could be applied to KSHV. Furthermore, this insight enhances our understanding of the general mechanisms of viral infection for the entire γ‐herpesvirus family.

## 4 Experimental Section

### Cell Line and Virus

Expi293F cells (ThermoFisher, Cat# A14527) were maintained in 293F medium (Union, Cat# UP1000) at 37  °C with 5% CO2 and shaking at 120 rpm. The iSLK.219 cells (gift from Prof. Lan) used for KSHV production, CNE2‐EBV cell line used for EBV production, and the HEK293 cells (ATCC, Cat# CRL‐11268) used for virus neutralization assay were cultured in DMEM (GIBCO, Cat# C11995500BT) with 10% FBS (ExCell Bio, Cat# FSP100) at 37 °C with 5% CO2. All mediums were supplemented with 100 IU mL⁻^1^ Penicillin/Streptomycin. All cell lines were regularly tested and confirmed to be free of mycoplasma contamination.

### Plasmid Construction

For the KSHV gB segment, spanning residues 1–653(Strain GK18, UniProtKB accession number: F5HB81), the sequence for mammalian expression was optimized to achieve the high‐efficiency translation required for recombinant protein expression, which was then synthesized and cloned into a mammalian expression vector. The vector features an N‐terminal Kozak sequence and a C‐terminal His‐tag for purification. Initial attempts to express the wild‐type protein were unsuccessful, prompting to modify the gB construct. This modification was inspired by analogous regions in the gB ectodomain of other herpesviruses,^[^ ^35^ ^]^ specifically replacing two putative FL regions, LT^128‐129^ and WFPGI ^209–212^, with sequence HR and RVEAT, respectively —adaptations borrowed from HSV‐1 gB^30^. In addition, the furin cleavage site RKRRS^437‐441^ was substituted with a flexible linker GGSGG, to create the modified KSHV gB653‐mut ectodomain.

KSHV gB residue 1–653 were cloned into a pCAGGS vector with N‐terminal Kozak sequence and C‐terminal 6*His‐tag. FL substitutions (LT ^128‐129^‐HR and WFPGI^209‐212^‐RVEAT) and furin site mutation (RKRRS^447‐451^‐GGSGG) were then introduced through homologous recombination. Briefly, primers containing the mutated site sequences and flanking homology arms were synthesized and used to amplify DNA fragments containing these mutated sites, with the wildtype KSHV gB residue 1–653 plasmid as template. Then, the amplified DNA fragments were cloned into pCAGGS plasmid digested with the *Eco*RI (NEB, Cat# R3101 V) and *Mlu*I (NEB, Cat# R3198 V) with Multis CE homologous recombination kit (Vazyme, Cat# C113‐01).

### Protein Expression and Purification

For protein expression, Expi293F cells, upon reaching a density of 1.5 × 10^6^/mL, were transiently transfected with the plasmid. The transfection mixture was prepared following a ratio of 1 mg plasmid to 4 mg Polyethylenimine Linear (PEI) MW40000 (Yeasen, Cat# 40816ES03) per 1 × 10^6^ cells/mL in 293F culture medium. This mixture was then added to the Expi293F cells. Five days post transfection, the supernatant was collected by centrifuge at 8 500 g for 1 h at 4 °C, and the cell debris was discarded. The supernatant was subsequently filtered through a 0.45 µm vacuum‐driven filter, and then incubated with Ni Sepharose resin (Cytiva, Cat# 17 371 202) for immobilized metal affinity chromatography. The resin was washed by the 10 column volumes of PBS for three times and eluted by 10 column volumes of PBS containing 300 mm imidazole. The eluate was then concentrated using a 30K MWCO ultracentrifuge tube (Millipore, Cat# UFC9030) and clarified by further centrifugation at 18 000 g for 10 min. The concentrated elution was applied to a Superdex 200 increase 10/300 GL size exclusion chromatography (SEC) column (Cytiva, Cat# 28 990 944) fitted on an AKTA Pure25m (Cytiva), using PBS as the SEC buffer. The fractions containing the target protein peaks were collected and subjected to ultrafiltration in a 30K MWCO ultracentrifuge tube. This step facilitated buffer exchange into PBS containing 0.1% DDM (n‐dodecyl β‐D‐maltoside). SEC analysis presented a single peak distribution (Figure S1A, Supporting Information), and the SDS‐PAGE showed a clear, single band (Figure S1B, Supporting Information), confirming the high purity and integrity of the KSHV gB653‐mut ectodomain as a monomeric protein. The protein was then stored at −80 °C.

Antibody purification was performed as previously described.^[^ ^59^ ^]^ Briefly, the transfection procedure was similar as above mentioned, and the supernatant was incubated with Protein A Sepharose resin (Cytiva, Cat# 17 127 902) and eluted with antibody elution buffer (0.2 m Glycine pH3.0). The elution was concentrated with 30K MWCO ultracentrifuge tube, and the solvent was gradually replaced by PBS after several rounds of ultracentrifuge. The concentrated antibody was then stored at 80 °C.

### Cryo‐EM Sample Preparation and Image Acquisition

Purified protein was checked by negative staining using the Talos L120C G2 (Thermo Fisher) at 120 kV. In brief, 4 µL of 0.5 mg mL⁻^1^ sample was loaded onto a freshly glow‐discharged Quantifoil Cu R1.2/1.3 300 mesh grids (Millipore, Cat# TEM‐Q350AR1.3‐2NM), plunge freezing was carried out using a Vitrobot Mark IV system (ThermoFisher), with 0‐blot force and 7 s blot time, 100% humidity and 8 °C. Cryo‐EM image data were acquired on a 300 kV Titan Krios (Thermo Fisher) equipped with a BioContinuum Imaging Filter (Gatan), and images were recorded on a K3 Summit direct detection camera (Gatan) using EPU software and GIF Quantum energy filter for automated image acquisition. The images were recorded at a nominal magnification of 105 000 × in super‐resolution mode, yielding a calibrated pixel size of 0.855 Å.

### Cryo‐EM Data Processing

Cryo‐EM data processing was performed with best practice of cryoSPARC (v3.3.2).^[^ ^60^ ^]^ Raw frames were dose‐weighted and corrected for beam‐induced drift by the motion correction. The contrast transfer function (CTF) parameters were estimated by CTFFIND4. Particles were first selected using blob picker, and further extracted using a box size of 384 pixels for 2D (2‐dimensions) classification. Representative 2D classes with different orientations were selected as templates for Topaz training. Then, Topaz extract was used to pick particles and subjected them to another round of 2D classification. After ab‐initio model building and homogeneous refinement, heterogeneous refinement was carried out. Specific class particles were used to nonuniform (NU) refinement. This KSHV gB detailed structure was derived from analyzing 172 723 particles, employing C3 symmetry in the 3D reconstruction process with a global resolution of 3.26 Å^[^ ^61^ ^]^ (Figure 1C; Figure S2, Supporting Information). Local refinement focused on the interface with the mask could reconstitute the structure at a 3.18 Å resolution. The structure of KSHV gB in complex with 2C4 fab was resolved at final resolution of 3.61 Å, and the local interface resolution is 2.92 Å. A local resolution estimate was performed with ResMap.^[^ ^62^ ^]^ Reported resolutions are based on the gold‐standard FSC of 0.143 criterion.

### Model Building, Refinement and Validation

The initial model of KSHV gB predicted by AlphaFold2^[^ ^63^ ^]^ was docked into the cryo‐EM density maps using Chimera.^[^ ^64^ ^]^ The models were manually corrected for local fit in COOT.^[^ ^65^ ^]^ The models were refined against corresponding maps in real space using PHENIX,^[^ ^66^ ^]^ in which the secondary structural restraints and Ramachandran restraints were applied. The stereochemical quality of each model was assessed using MolProbity.^[^ ^67^ ^]^ The Q‐scores were calculated to estimate the quality of model‐to‐map^[^ ^68^ ^]^ (Figures S4,S10,S11, Supporting Information). Statistics for model refinement and validation are shown in Table S1 (Supporting Information).

### Structural Prediction of the Wild‐Type KSHV gB

AlphaFold2 was used to predict the wild‐type KSHV gB 1–653. Briefly, unlike the initial model generated for building an atomic model based on cryo‐EM data, the AlphaFold2 model for wild‐type KSHV gB was created with the resolved structure in this study as template to constraint model generation. The pLDDT (per‐residue local‐distance difference test) score was used to evaluate the prediction accuracy at residue level.

### Multiple Sequence Alignment and Phylogenetic Tree Building

To assess the global sequence homology across herpesviruses, protein sequence from UniProtKB of KSHV gB (Strain GK18, Accession number: F5HB81) to HSV‐1 (Strain KOS, Accession number: P06437), VZV (Strain Oka, Accession number: Q4JR05), HCMV (Strain AD169, Accession number: P06437), and EBV (Strain B95‐8, Accession number: P03188) were retrieved. Then, ClustalW multiple sequence alignment (MSA) (https://www.genome.jp/tools‐bin/clustalw) was performed to calculate sequence similarity and identity. The alignment result was further processed using ESpript3.X (https://espript.ibcp.fr/ESPript/cgi‐bin/ESPript.cgi) to visualize the alignment.

To assess the sequence homology of herpesviruses gB with available structural information, ENDscript2.X (https://endscript.ibcp.fr/ESPript/cgi‐bin/ENDscript.cgi) was used to search the reported structures with homologous sequences to KSHV gB, and MSA was performed with Clustal Omega to generate alignment results and visualize the alignment. Based on the MSA result, Phylodendron (http://iubioarchive.bio.net/treeapp/) was used to generate the phylogenetic tree with distance.

### Biolayer Interferometry

Biolayer interferometry (BLI) assay was performed to determine the cross‐herpesviruses antigenicity of gB. Briefly, gB ectodomains of different human herpesvirus, including HSV‐1, VZV, HCMV and EBV, and the corresponding neutralizing antibodies targeting these gB, including D48, 93K, SM5‐1, 3A3, 3A5, and Fab5 were prepared as previously reported. The newly characterized EBV gB neutralizing antibody Fab5 was identified in the lab as unpublished data. The dilution series followed a twofold progression, ranging from 500 to 31.25 nm. Protein A sensors (Sartorious, Cat# 18–5010) were used to capture antibody at 10 µg mL⁻^1^. After equilibration in KB buffer (PBS, 0.1% BSA, 0.02% Tween), the sensors with captured antibody were associated to 500 nm of each gB for 100 s, followed by a dissociation phase in KB buffer for 100 s.

### Virus Production and Neutralization Assay

To produce EBV, CNE2‐EBV cells were induced with 20 ng mL⁻^1^ 12‐O‐Tetradecanoylphorbol 13‐acetate (TPA, Beyotime, Cat# S1819) and 2.5 mm Sodium butyrate (NaB, Beyotime, Cat# S1539) in fresh RPMI1640 medium for 12 h. The medium was then replaced with the regular culture medium for 60 h culture for EBV production. The supernatant was then collected and filtered through a 0.45 µm filter cup (JET bio, Cat# FPE204000). The virus was concentrated by centrifugation at 50 000 g at 4 °C for 3 h, and the virus pellet was resuspended in RPMI1640 and stored at −80 °C. KSHV was prepared as previously described. Briefly, iSLK.219 cells harboring rKSHV.219 were induced with 1 mg mL⁻^1^ doxycycline (MCE, Cat# HY‐N0565B) and 1 mm NaB for 5 days. The supernatant was centrifuged at 4 000 g for 30 min at 18 °C to remove cell debris, followed by a second centrifugation at 80 000 g for 2 h at 4 °C. The virus pellet was then resuspended with fresh RPMI1640 and stored at –80 °C until use.

HEK293 cells were susceptible to both EBV and KSHV and used for neutralization assay. Briefly, 30 000 HEK293 cells per well were seeded into 96‐well plates and cultured in DMEM containing 10% FBS at 37 °C and 5% CO_2_ overnight. Antibodies including 3A3, 3A5, Fab5, and control IgG were diluted in DMEM to achieve a final concentration series as 5, 2.5, 1.25, 0.62, 0.31, 0.16, 0.08, 0.04, 0.02 µg mL⁻^1^ for EBV and 200, 100, 20, 4, 0.8, 0.16, 0.03, 0.006, 0.0012 µg mL⁻^1^ for KSHV. These antibody solutions were then thoroughly mixed with prepared EBV or KSHV, adjusting the antibody concentration in the antibody‐virus mixtures to 50 µg mL⁻^1^ and incubated at 37 °C for 1 h. Subsequently, the supernatant in the cell culture wells of the 96‐well plate was removed, and 60 µL of the antibody‐virus mixture was added to each well.

Additionally, blank wells were set up by adding 60 µL of DMEM as an uninfected control. After the addition, the plates were incubated at 37 °C for 1 h, followed by the addition of 120 µL of DMEM containing 10% FBS per well. The cells were cultured for 48 h and then digested with trypsin (NCM, Cat# C100C1) for flow cytometry analysis. The percentage of GFP‐positive cells, indicative of EBV or KSHV infection, was determined using uninfected cells as negative controls. The normalized EBV or KSHV infection was calculated by GFP + % of each well/mean GFP + % of control IgG group. The IC50 was calculated using non‐linear fitting (four parameters) in GraphPad Prism 10.0.

## Conflict of Interest

The authors declare no conflict of interest.

## Author Contributions

X.Y.F., C.S., and C.X. Contributed equally to this work. C.S., M.S.Z., S.F.S., and Z.L. designed the work. X.Y.F. and B.Z.C performed Cryo‐EM image data acquisition. X.Y.F. performed Cryo‐EM data processing and model building. C.S. and X.Y.F. analyzed structure. C.S., C.X., and G.X.Z. performed protein purification, binding assays, and neutralization assays. Z.Z.L. provided support for antibody validation and virus preparation. C.S., X.Y.F., and Z.L. wrote the manuscript. All authors contributed to the completion of this manuscript.

## Supporting information



Supporting Information

## Data Availability

The cryo‐EM structure and density map of KSHV gB have been deposited in PDB under accession number 8Y48 and EMDB under accession number EMD‐38910. The cryo‐EM structure and density map of KSHV gB‐2C4 fab have been deposited in PDB under accession number 9LLD and EMDB under accession number EMD‐63198. The local map of KSHV gB‐2C4 Fab interface's accession number is EMD‐63209. Materials and plasmids are available in request to corresponding authors.
