# Supplementary material for: Structure and Antigenicity of Kaposi's Sarcoma‐Associated Herpesvirus Glycoprotein B
Source: Adv Sci (Weinh). 2025 Apr 26;12(27):2502231. doi: 10.1002/advs.202502231 (PMC12279188; doi:10.1002/advs.202502231)
Supplement: Supplementary file 1 — Supporting Information [file ADVS-12-2502231-s001.pdf]

ADVANCED SCIENCE
Open Access

Supporting Information

for *Adv. Sci.*, DOI 10.1002/advs.202502231

Structure and Antigenicity of Kaposi's Sarcoma-Associated Herpesvirus Glycoprotein B

Xin-Yan Fang, Cong Sun, Chu Xie, Bing-Zhen Cheng, Zheng-Zhou Lu, Ge-Xin Zhao, Sen-Fang Sui*, Mu-Sheng Zeng* and Zheng Liu**

Supporting Information

Structure and antigenicity of Kaposi's sarcoma-associated herpesvirus glycoprotein B

Xin-Yan Fang[1,2,5], Cong Sun[3,5,6], Chu Xie[3,5], Bing-Zhen Cheng[1,2], Zheng-Zhou Lu[3], Ge-Xin Zhao[3,4], Sen-Fang Sui[1,2,6], Mu-Sheng Zeng[3,6], Zheng Liu[1,6]

[1] Cryo-electron Microscopy Center, Southern University of Science and Technology, Shenzhen, Guangdong, 518055, China

[2] Department of Biology, Southern University of Science and Technology, Shenzhen, Guangdong, 518055, China

[3] State Key Laboratory of Oncology in South China, Guangdong Provincial Clinical Research Center for Cancer, Guangdong Key Laboratory of Nasopharyngeal Carcinoma Diagnosis and Therapy, Sun Yat-sen University Cancer Center, Guangzhou 510060, P. R. China.

[4] Department of Dermatology, Vagelos College of Physicians and Surgeons, Columbia University, New York, NY, USA.

[5] These authors contributed equally to this work.

[6]Corresponding authors

Email: liuz3@sustech.edu.cn

Email: zengmsh@sysucc.org.cn

Email: suisf@tsinghua.edu.cn

Email: suncong@sysucc.org.cn

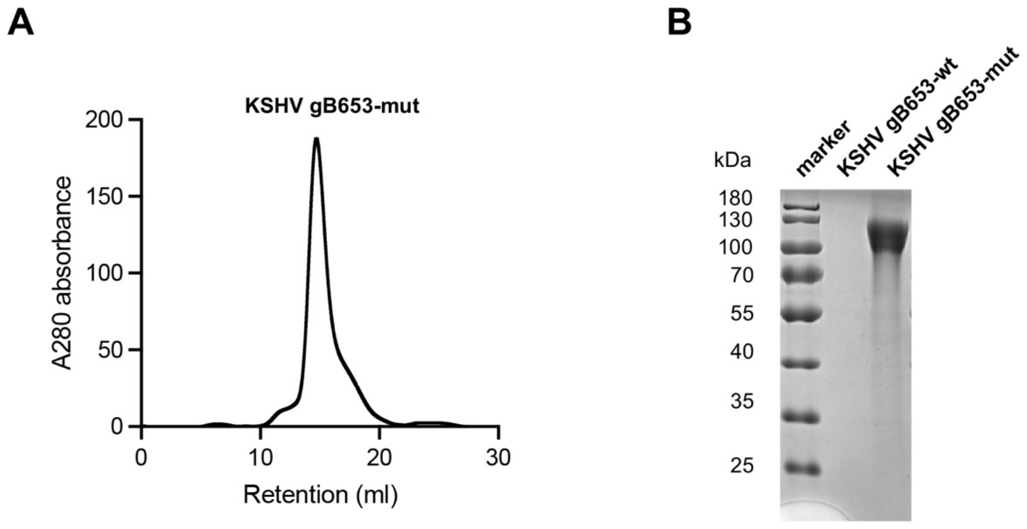

Figure S1. Biochemical characterization for the KSHV gB653-mut ectodomain.

(A). Size exclusion chromatography (SEC) analysis of KSHV gB653-mut using the Superdex200 Increase 10/300GL column.

(B). SDS-PAGE analysis of the KSHV gB653-wt (wild-type construction) and KSHV gB653-mut following initial purification.

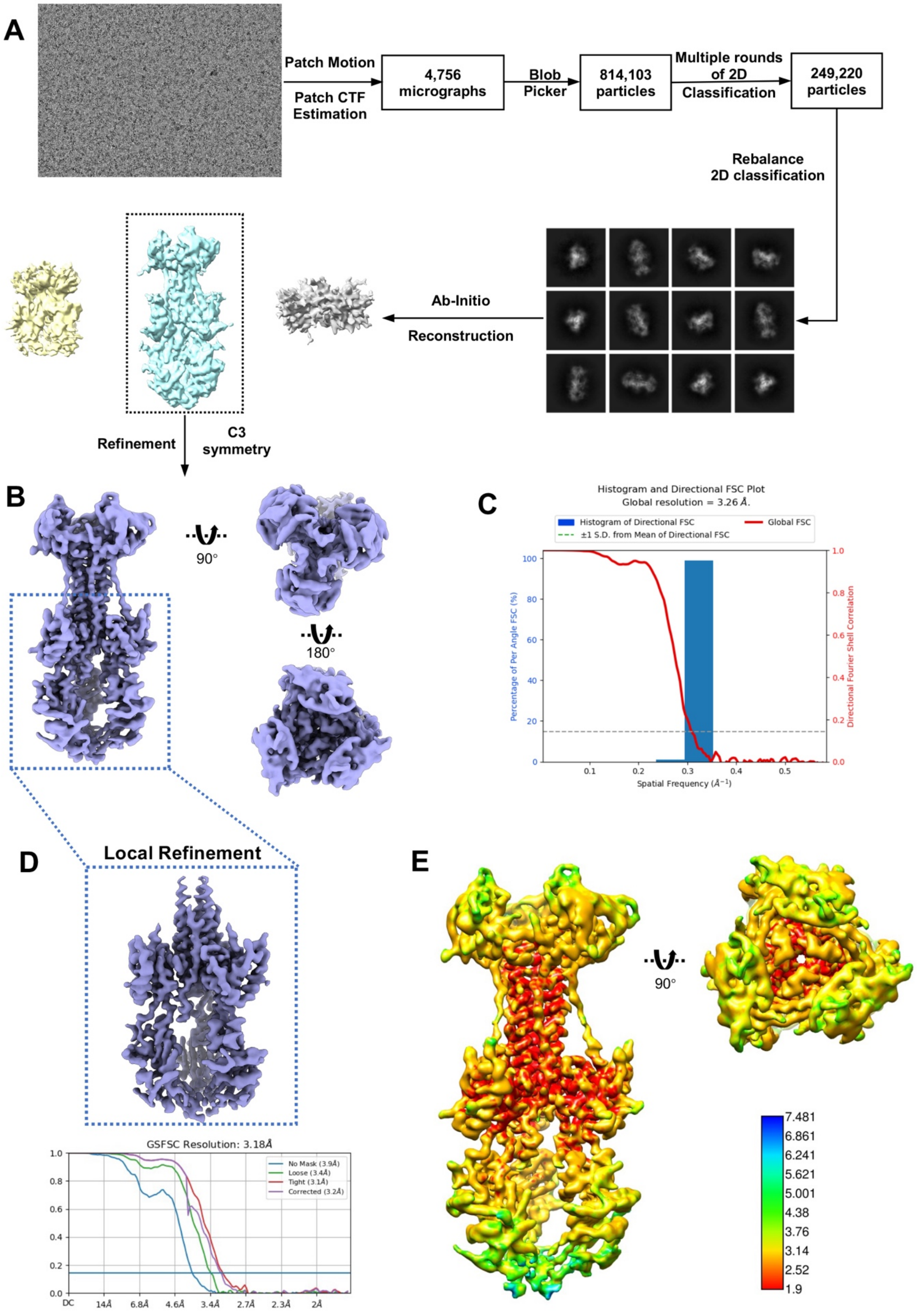

Figure S2. Cryo-EM data processing and 3D reconstruction analysis workflow.

(A). Workflow for image processing.

(B). Three views of the NU refinement KSHV gB map.

(C). 3D Fourier Shell Correlation (FSC) analysis of NU refinement KSHV gB map. Gold-standard FSC curve with indicated resolution at 0.143.

(D). Local refinement map of KSHV gB DI and DII with FSC curves. Gold-standard FSC curve between two half maps is shown with indicated resolution at 0.143.

(E). Local resolution map of KSHV gB.

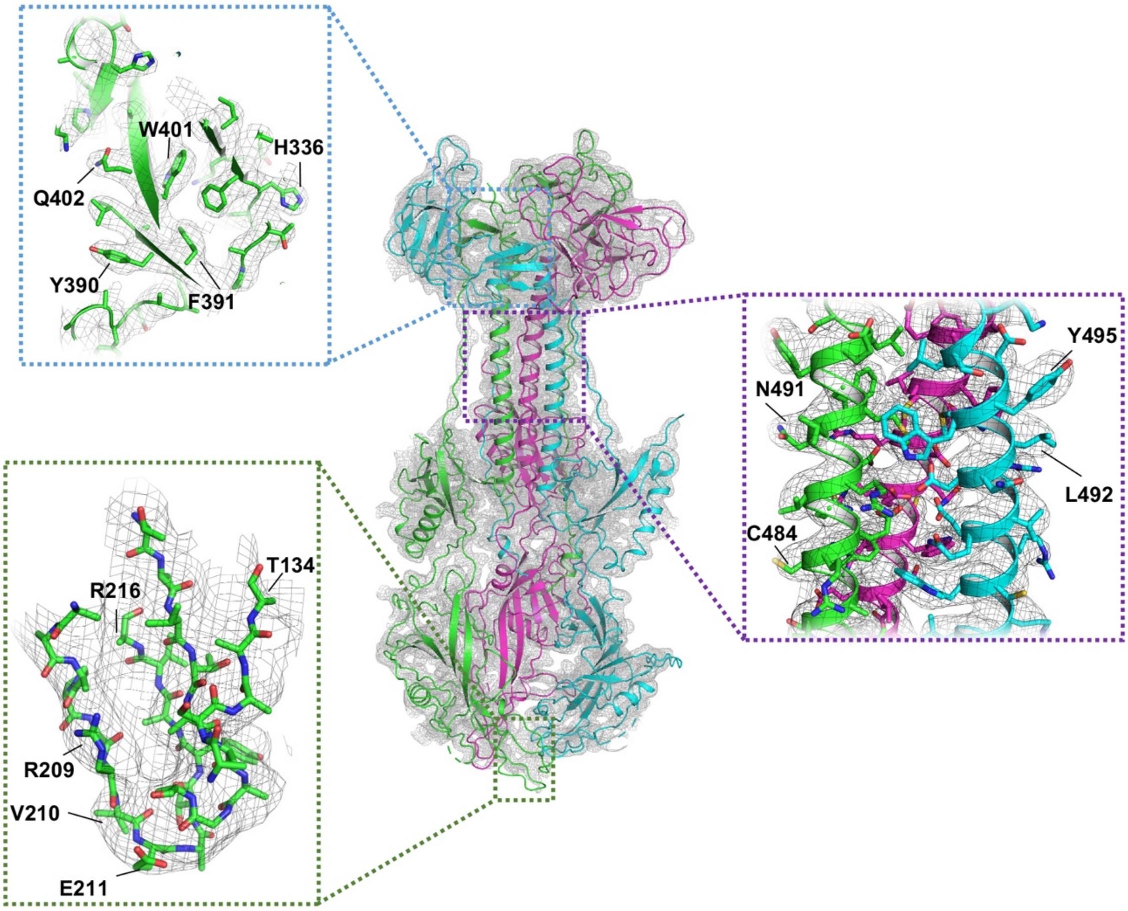

Figure S3. Fitting the model to the density map.

The cryo-EM density map is shown as mesh, with atomic models shown as cartoon with each monomer colored differently. Key residues and structural elements are labeled.

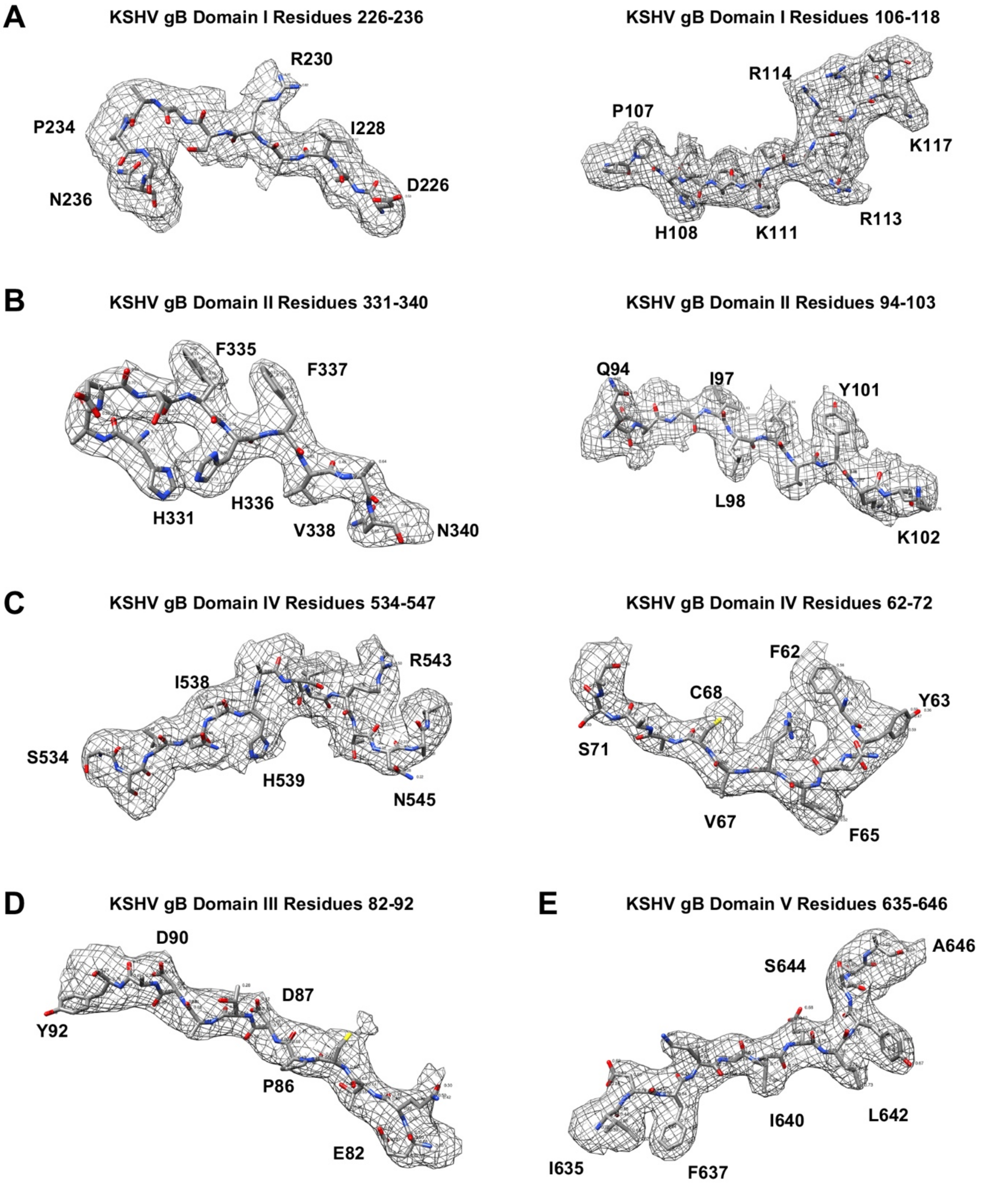

Figure S4. The model-to-map and corresponding Q scores of KSHV gB domains I-V.

Atomic models of eight representative peptides are shown in sticks. The corresponding atomic Q scores are labeled.

(A). Domain I (Residues 106-118 and 226-236),

(B). Domain II (Residues 331-340 and 94-103),

(C). Domain IV (Residues 534-547 and 62-72),

(D). Domain III (Residues 82-92),

(E). Domain V (Residues 635-646).

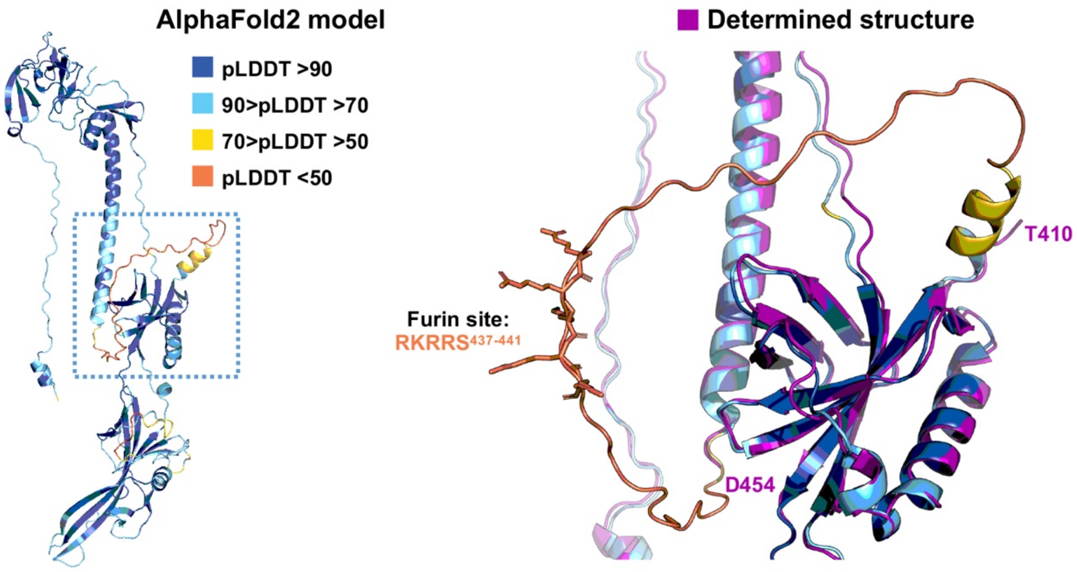

Figure S5. AlphaFold2 prediction of KSHV gB653-wt.

The AlphaFold2 model is displayed as a cartoon, colored according to the pLDDT score. Zoomed in view shows the structural alignment results between the experimentally determined model (colored purple) and the AlphaFold2 model. The starting and ending residues of the missing region in the determined structure are labeled. The furin cleavage site RKRRS in the AlphaFold2 model is depicted as sticks.

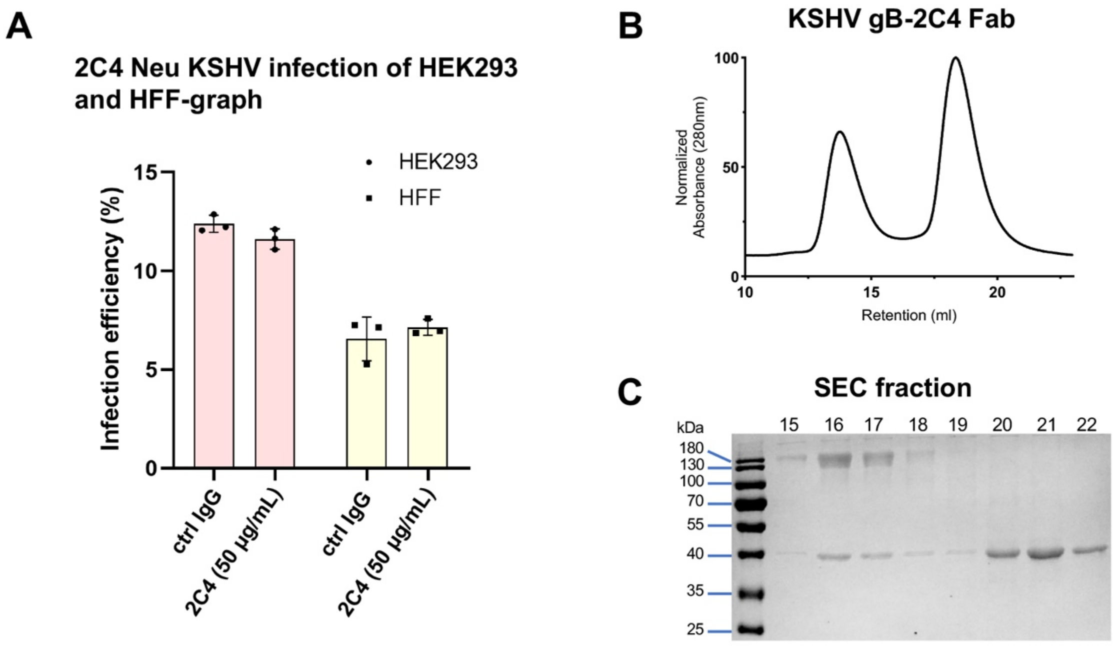

Figure S6. Evaluation of 2C4 IgG for neutralization of KSHV infection and Biochemical characterization for the complex of KSHV gB653-mut ectodomain and 2C4 Fab.

(A). Evaluation of 2C4 IgG for neutralization of KSHV infection in HEK293 and HFF cells. KSHV was pre-incubated with either control IgG (Ctrl IgG) or the 2C4 IgG antibody at 50 µg/mL before being added to HEK293 (epithelial) and HFF (fibroblast) cells for infection. Infection efficiency was quantified as the percentage of infected cells (GFP positive). Each data point represents the mean of technical replicates from a single experiment, and error bars indicate standard deviation. This experiment assesses whether 2C4 IgG exhibits neutralizing activity against KSHV infection in these cell types.

(B). Size exclusion chromatography (SEC) analysis of the complex using the Superdex200 Increase 10/300GL column.

(C). SDS-PAGE analysis of the complex following initial purification.

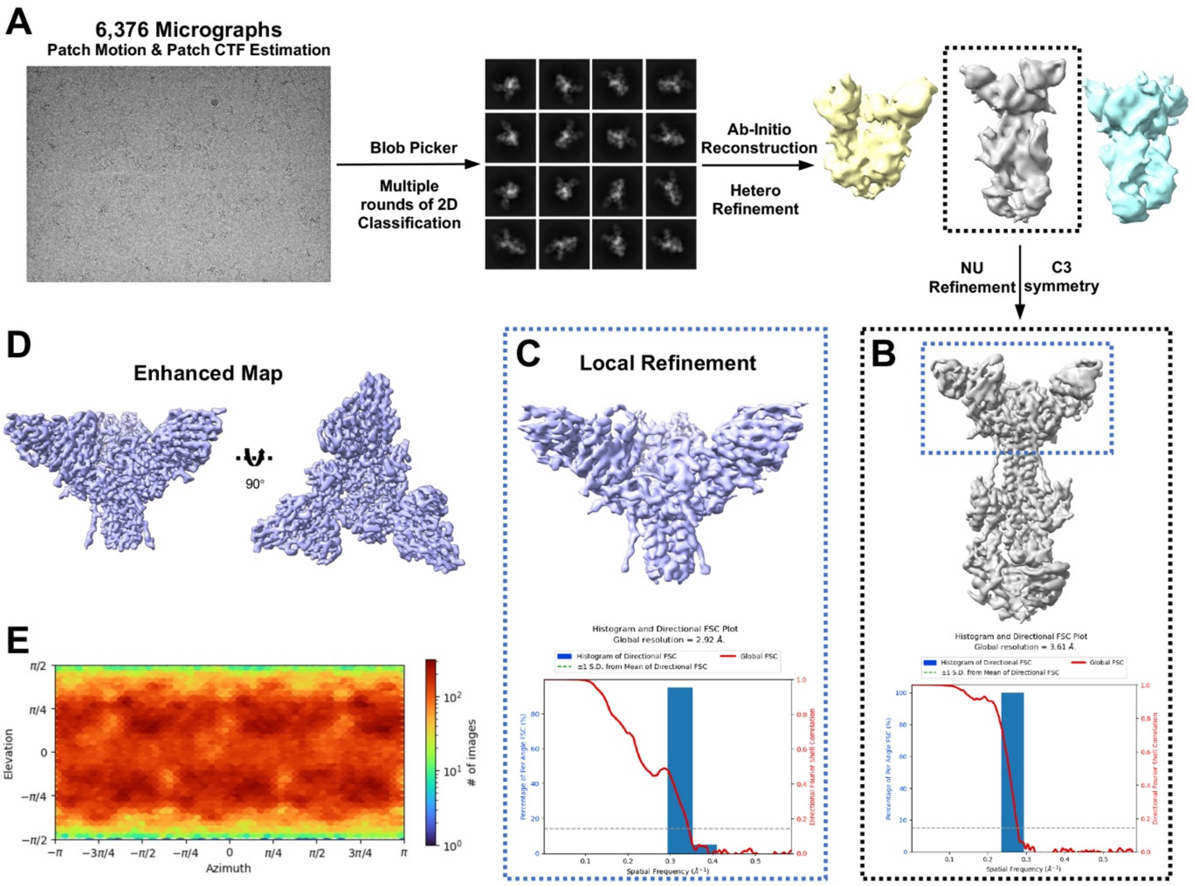

Figure S7. Cryo-EM data processing and 3D reconstruction analysis workflow of KSHV gB in complex with 2C4 Fab.

(A). Workflow for image processing.

(B). NU refinement map and 3D FSC of KSHV gB–2C4 Fab complex, showing the gold-standard FSC curve with indicated resolution marked at 0.143.

(C). Local refinement map and its 3D FSC of KSHV gB DIV–2C4 Fab interface, showing the gold-standard FSC curve between two half maps with resolution indicated at 0.143.

(D). Local map of KSHV gB DIV–2C4 Fab interface by EMready.

(E). Viewing direction distribution of KSHV gB–2C4 Fab.

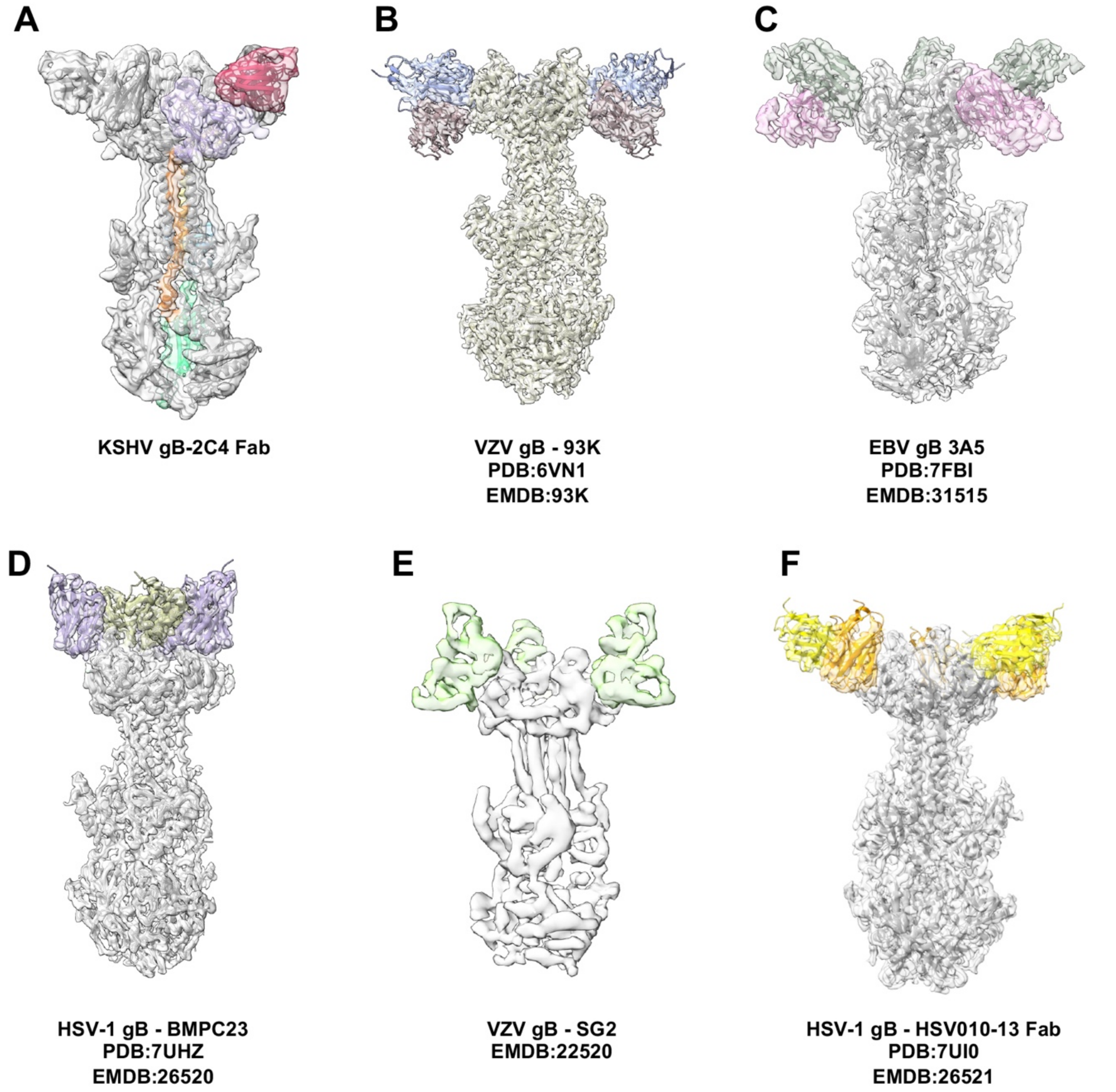

Figure S8. Various epitopes on Domain IV targeted by herpesvirus gB-specific antibodies.

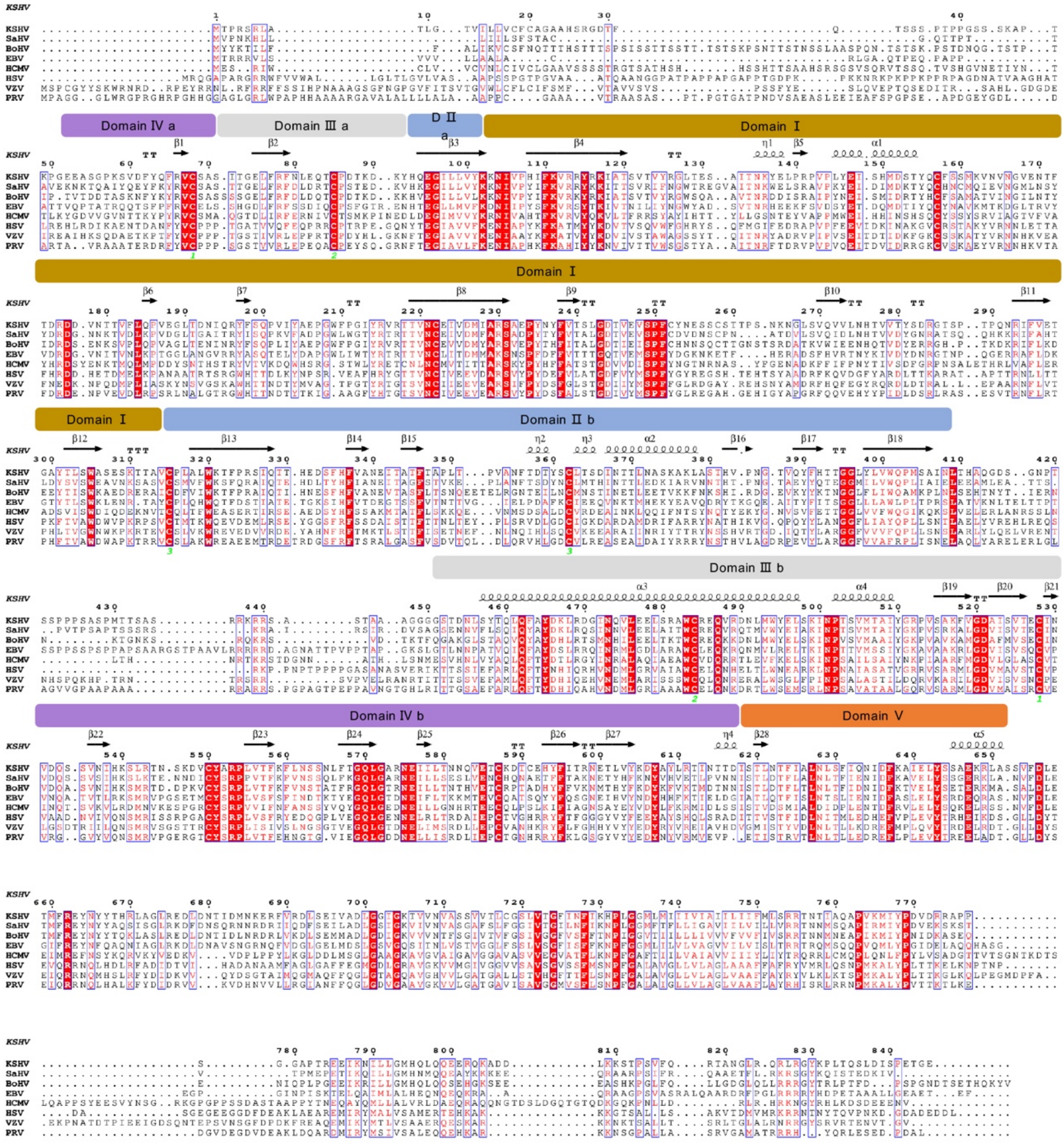

Figure S9. Multiple sequence alignment of KSHV, SaHV, BoHV, VZV, EBV, HCMV, HSV, VZV, and PRV.

The red boxes highlight identical residues, while the blue boxes outline similar residues in the alignment.

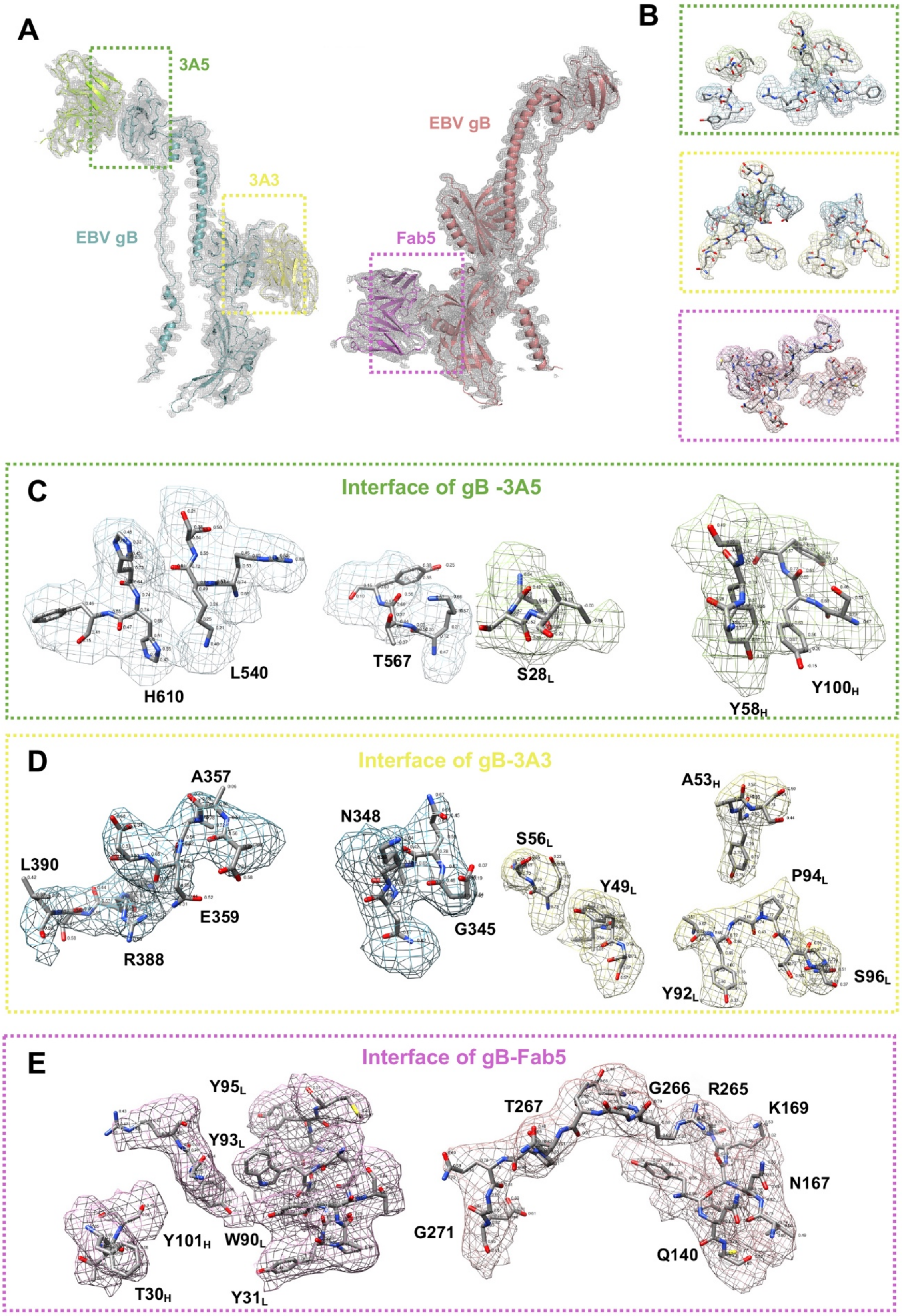

Figure S10. The cryo-EM maps (mesh) of EBV gB-3A3, 3A5, and Fab5

superimposed with corresponding atomic models.

(A). Density maps of EBV gB-3A3, 3A5 (left) (PDB:7FBI; EMD-31515) and EBV gB-Fab5 (right) (PDB:8YY6; EMD-39670).

(B). Enlarged boxes represent the interfaces density of gB-3A3 (top), gB-3A5 (middle) and gB-Fab5 (bottom).

(C-E). Q scores were calculated to estimate the quality of model-to-map of the residues on the EBV gB-mAb interface. Three neutralizing antibodies 3A5 (C), 3A3 (D) and Fab5 (E) are colored in pale green, khaki, and plum dash boxes, respectively.

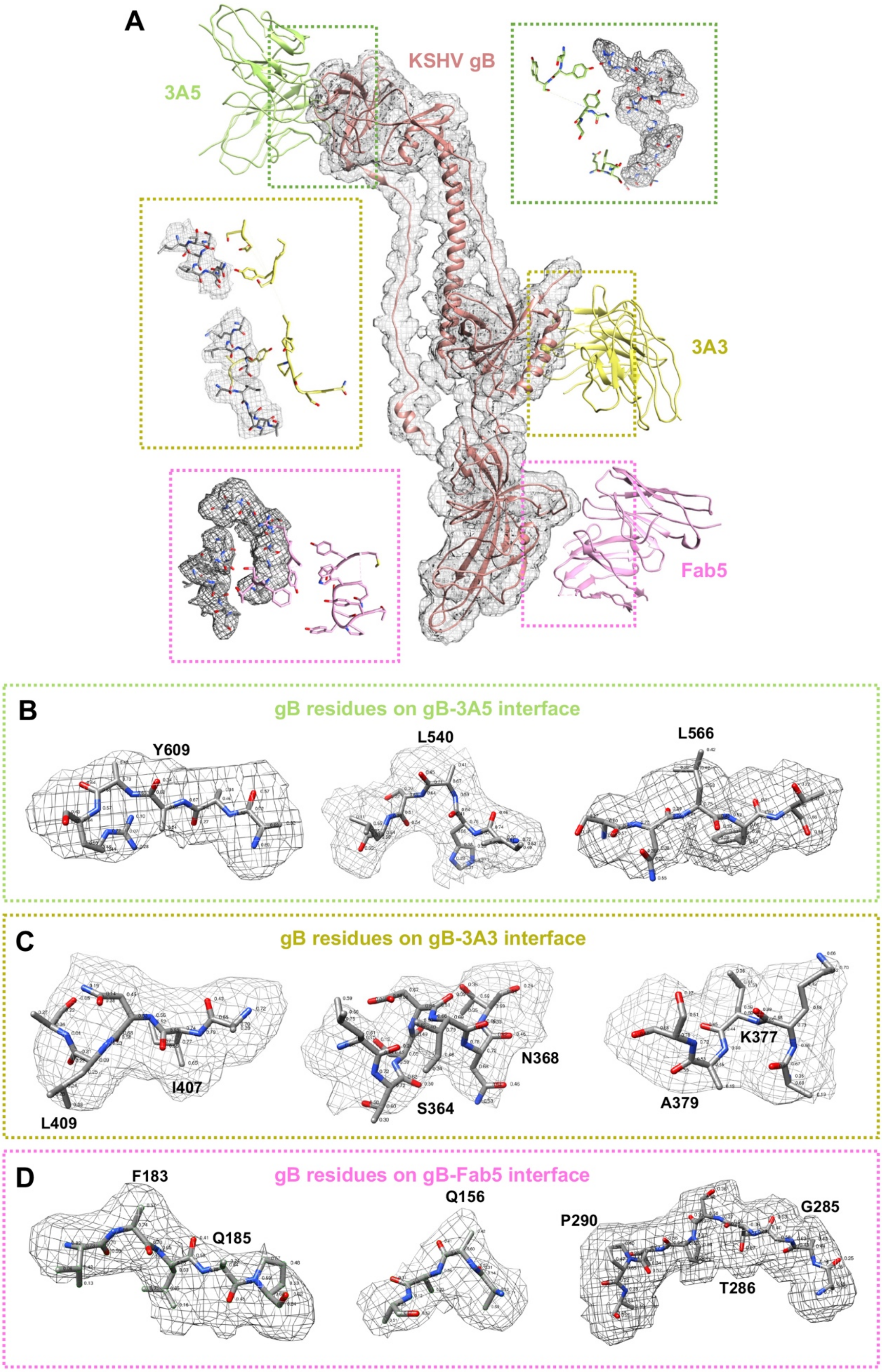

Figure S11. Structural fitting and predicted interfaces of KSHV gB with

neutralizing antibodies 3A5, 3A3, and Fab5.

(A). Overall density map of KSHV gB (pink) with the predicted positions of three neutralizing antibodies: 3A5 (pale green), 3A3 (khaki), and Fab5 (plum). The three possible interfaces were predicted through structural alignment between EBV gB-mAb (PDB:7FBI for 3A3, 3A5 and PDB:8YY6 for Fab5) and KSHV gB (in this study) based on gB structure. The predicted interface regions between KSHV gB and 3A5, 3A3, and Fab5 are highlighted in pale green (zoom 1), khaki (zoom 2), and plum (zoom 3) dashed boxes.

(B-D). Residues of KSHV gB likely involved in interactions with the antibodies 3A5 mAb (B), 3A3 mAb (C), and Fab5 mAb (D). Residues are shown in stick, with atomic Q scores were calculated to estimate the model-to-map quality.

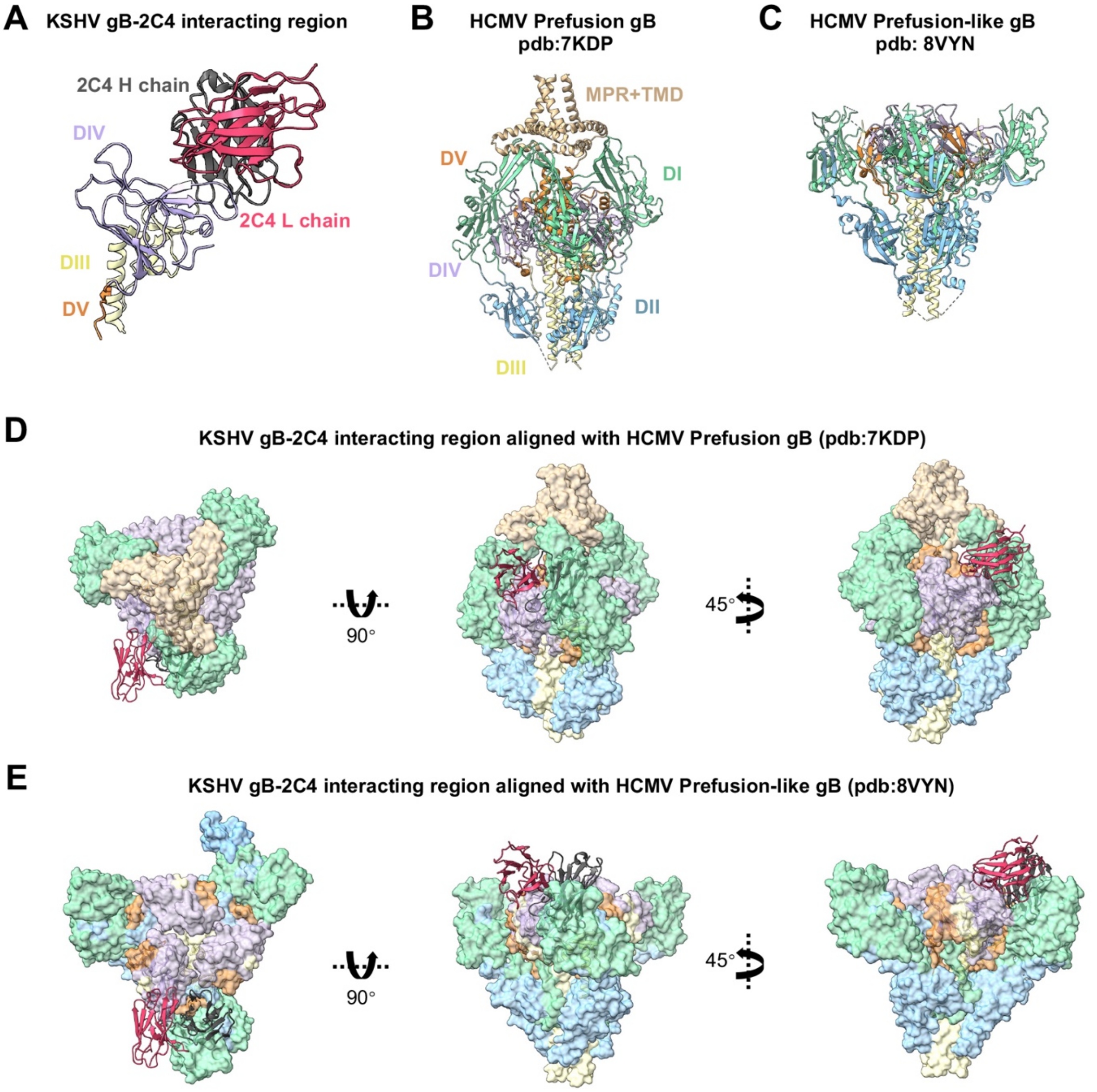

Figure S12. Superimposition of the KSHV gB-2C4 interacting region with HCMV gB in prefusion state.

(A). Structure of KSHV gB-2C4 interacting region.

(B). Structure of HCMV prefusion gB (pdb: 7KDP), with DI-DV colored as Figure 1.

(C). Structure of HCMV prefusion-like gB (pdb: 8VYN).

(D). Three views of superimposition of the KSHV gB-2C4 interacting region with HCMV prefusion gB (pdb: 7KDP) based on DIV alignment.

(E). Three views of superimposition of the KSHV gB-2C4 interacting region with HCMV prefusion gB (pdb: 8YVN) based on DIV alignment.

Table S1. Cryo-EM data collection, refinement, and validation statistics

	KSHV gB (PDB: 8Y48 EMD-38910)	KSHV gB-2C4 Fab (PDB: 9LLD EMD-63198)	Local map of KSHV gB-2C4 Fab Interface (EMD-63209)
**Data collection and processing**			
Microscope	Titan Krios	Titan Krios	Titan Krios
Camera	Gatan K3	Gatan K3	Gatan K3
Magnification	105,000	105,000	105,000
Voltage(kV)	300	300	300
Electron exposure (e^{-}/Å^2)	50	50	50
Defocus range (μm)	-1.0 ~ -2.0	-1.0 ~ -2.0	-1.0 ~ -2.0
Pixel size (Å)	0.855	0.855	0.855
Frames/movie	32	32	32
Movies (total)	4,756	6376	6376
Initial particle images (no.)	814,103	680,579	680,579
Final particle images (no.)	120,100	339379	339379
Symmetry imposed	C3	C3	C3
**Map resolution (Å)**			
FSC threshold	0.143	0.143	0.143
Map resolution range (Å)	1.9 – 7.481	2-6.565	
**Model composition**			
Non-hydrogen atoms	12051	16287	
Protein residues	1674	2334	
Ligands	0	0	
**R.m.s. deviations**			
**Bond lengths (Å)**	0.002	0.005	
Bond angles (°)	0.477	0.963	
**Validation**			
MolProbity score	1.75	1.89	
Clashscore	6.70	8.69	
Rotamers outliers (%)	0	0.07	
**Ramachandran plot**			
Favored (%)	94.40%	6.32%	
Allowed (%)	5.60%	93.68%	
Disallowed (%)	0	0	

Table S2. Kinetic data for BLI assays calculated using Octet Analysis Studio.

Loading antibody	Sample gB	KD (M)	KD Error	ka (1/Ms)	ka Error	kdis (1/s)	kdis Error
D48	HSV-1	2.720E-11	4.849E-10	2.465E04	2.539E02	6.705E-04	9.755E-06
D48	VZV	1.000E-06	0.000E00	1.000E04	0.000E00	1.000E-03	0.000E00
D48	HCMV	1.000E-06	0.000E00	1.000E04	0.000E00	1.000E-03	0.000E00
D48	EBV	1.000E-06	0.000E00	1.000E04	0.000E00	1.000E-03	0.000E00
D48	KSHV	1.000E-06	0.000E00	1.000E04	0.000E00	1.000E-03	0.000E00
93K	HSV-1	1.000E-06	0.000E00	1.000E04	0.000E00	1.000E-03	0.000E00
93K	VZV	3.869E-12	2.372E-09	1.402E05	3.349E02	5.426E-07	1.548E-04
93K	HCMV	1.229E-06		6.929E04	4.486E03	8.519E-04	
93K	EBV	1.000E-06	0.000E00	1.000E04	0.000E00	1.000E-03	0.000E00
93K	KSHV	1.000E-06	0.000E00	1.000E04	0.000E00	1.000E-03	0.000E00
SM5-1	HSV-1	1.808E-06		3.318E05	1.199E04	5.998E-03	1.667E-04
SM5-1	VZV	1.000E-06	0.000E00	1.000E04	0.000E00	1.000E-03	0.000E00
SM5-1	HCMV	1.714E-11	8.243E-10	4.389E04	8.286E02	7.521E-07	2.207E-04
SM5-1	EBV	1.000E-06	0.000E00	1.000E04	0.000E00	1.000E-03	0.000E00
SM5-1	KSHV	1.000E-06	0.000E00	1.000E04	0.000E00	1.000E-03	0.000E00
3A3	HSV-1	1.678E-06	1.334E-06	1.544E03	1.228E03	2.591E-03	5.189E-05
3A3	VZV	1.000E-06	0.000E00	1.000E04	0.000E00	1.000E-03	0.000E00
3A3	HCMV	1.000E-06	0.000E00	1.000E04	0.000E00	1.000E-03	0.000E00
3A3	EBV	5.047E-12	2.442E-09	2.197E05	1.223E03	1.109E-06	8.948E-05
3A3	KSHV	1.017E-06		4.803E03	2.010E04	4.883E-07	
3A5	HSV-1	4.515E-07	3.184E-07	1.464E03	1.028E03	6.608E-04	4.125E-05
3A5	VZV	1.000E-06	0.000E00	1.000E04	0.000E00	1.000E-03	0.000E00
3A5	HCMV	4.573E-06	1.227E-06	6.605E02	1.763E03	3.021E-04	7.935E-05
3A5	EBV	2.330E-12		4.410E05	4.718E03	1.027E-06	
3A5	KSHV	2.064E-06		2.366E03	7.265E03	4.883E-07	
Fab5	HSV-1	1.771E-06	2.583E-08	9.130E03	1.298E03	1.617E-03	5.264E-05
Fab5	VZV	1.000E-06	0.000E00	1.000E04	0.000E00	1.000E-03	0.000E00
Fab5	HCMV	2.876E-07		1.459E04	8.164E02	4.195E-04	
Fab5	EBV	2.855E-12	2.705E-09	3.069E05	2.569E03	8.761E-07	3.171E-05
Fab5	KSHV	2.068E-08	7.370E-10	1.464E05	4.188E03	3.028E-04	1.076E-04

Table S3. Antibody neutralization assay data for EBV infection in HEK293 cells.

EBV			
	**3A3**	**3A5**	**Fab5**
log(agonist) vs. response -- Variable slope (four parameters)			
Best-fit values			
Bottom	-5.825	-18.01	-2.245
Top	100.9	102.7	115.9
LogEC50	-0.4009	0.127	-0.7699
HillSlope	-1.093	-0.9026	-0.9784
EC50	0.3973	1.34	0.1699
Span	106.8	120.7	118.2
95% CI (profile likelihood)			
Bottom	-11.69 to -1.233	-101.0 to 2.055	-10.47 to 3.517
Top	95.44 to 108.3	94.40 to 119.6	104.1 to 138.4
LogEC50	-0.4773 to -0.3301	-0.08323 to 0.7835	-0.9499 to -0.6504
HillSlope	-1.319 to -0.8890	-1.366 to -0.4748	-1.275 to -0.7071
EC50	0.3332 to 0.4676	0.8256 to 6.074	0.1122 to 0.2237
Goodness of Fit			
Degrees of Freedom	36	36	36
R squared	0.9868	0.9407	0.9743
Sum of Squares	753.7	3250	1468
Sy.x	4.576	9.501	6.385
Number of points			
# of X values	40	40	40
# Y values analyzed	40	40	40

Table S4. Antibody neutralization assay data for KSHV infection in HEK293 cells.

KSHV			
	**3A3**	**3A5**	**Fab5**
log(agonist) vs. response -- Variable slope (four parameters)			
Best-fit values			
Bottom			15.47
Top			99.46
LogEC50			0.1673
HillSlope			-0.5506
EC50			1.47
Span			85.74
95% CI (profile likelihood)			
Bottom			8.328 to 20.27
Top			93.41 to 115.1
LogEC50			-1.014 to 0.368
HillSlope			-0.7405 to -0.3837
EC50			0.09676 to 2.3335
Goodness of Fit			
Degrees of Freedom			32
R squared			0.9697
Sum of Squares			999.7
Sy.x			5.589
Number of points			
# of X values			36
# Y values analyzed			36
